# ZNF33B facilitates Japanese encephalitis virus replication by controlling HSPB1/8-mediated SUMOylation of nonstructural protein 5

**DOI:** 10.1128/jvi.00868-25

**Published:** 2025-09-08

**Authors:** Jian Du, Chunwei Li, Jinyan Zhang, Jiyuan Luo, Huizhi Zhang, Shengsong Xie, Huanchun Chen, Xiangmin Li, Ping Qian

**Affiliations:** 1 National Key Laboratory of Agricultural Microbiology, Hubei Hongshan Laboratory, Huazhong Agricultural University, Wuhan, China; 2 The Cooperative Innovation Centre for Sustainable Pig Production, Huazhong Agricultural University 47895, Wuhan, China; 3 College of Veterinary Medicine, Huazhong Agricultural University 47895, Wuhan, China; 4 Key Laboratory of Agricultural Animal Genetics, Breeding and Reproduction of Ministry of Education, Key Lab of Swine Genetics and Breeding of Ministry of Agriculture and Rural Affairs, Huazhong Agricultural University, Wuhan, China; 5 Hubei Jiangxia Laboratory, Wuhan, China; Loyola University Chicago - Health Sciences Campus, Maywood, Illinois, USA

**Keywords:** Japanese encephalitis virus, NS5, ZNF33B, SUMOylation

## Abstract

Japanese encephalitis virus (JEV) is a significant flavivirus that poses a threat to public health, as it induces encephalitis in humans and reproductive disorders in sows. We have recently identified that zinc finger protein 33B (ZNF33B) is required for JEV infection by CRISPR-based functional genomic screening, yet the precise functions and mechanisms are not fully comprehended. In this study, ZNF33B was found to be involved in JEV infection, wherein it bound with JEV RNA to enhance its stability during replication. Additionally, ZNF33B underwent translocation from the nucleus to the cytoplasm to associate with viral replication complexes during JEV infection. Furthermore, ZNF33B stabilized JEV nonstructural protein 5 (NS5), rather than NS3, by inhibiting its polyubiquitination and promoting SUMOylation. The SUMOylation of JEV NS5 was found to compete with its ubiquitination at lysine residues 269 and 846. Through immunoprecipitation-mass spectrometry, we identified heat shock proteins HSPB1 and HSPB8 as potential mediators of the SUMOylation of JEV NS5. ZNF33B was shown to recruit HSPB1/8 to facilitate NS5 SUMOylation. Overall, our study highlighted the importance of ZNF33B in facilitating the SUMOylation of JEV NS5 through the recruitment of HSPB1 and HSPB8.

**IMPORTANCE:**

Japanese encephalitis virus (JEV) poses a severe global health threat, yet host factors regulating its replication remain poorly understood. Our study identifies ZNF33B as a critical host protein that enhances JEV replication by stabilizing viral RNA and facilitating SUMOylation of the viral polymerase NS5. We demonstrate that ZNF33B recruits HSPB1/8 as SUMO E3 ligases to modify NS5, thereby counteracting its polyubiquitination and proteasomal degradation. This SUMOylation-ubiquitination crosstalk at lysine residues 269 and 846 ensures NS5 stability, essential for viral replication. These findings unveil a novel mechanism by which JEV exploits host post-translational machinery to sustain replication. Targeting ZNF33B or viral SUMOylation could offer therapeutic strategies against JEV and related flaviviruses, with great significance for the development of antiviral interventions.

## INTRODUCTION

Japanese encephalitis virus (JEV) is a member of the genus *Flavivirus* within the family *Flaviviridae*, which encompasses over 70 species, including West Nile virus, dengue virus, Zika virus (ZIKV), and yellow fever virus, and causes encephalitis in humans and reproductive disorders in pigs (1, 2). Although the vast majority of JEV-infected individuals do not have obvious symptoms, a small number of infected cases can still develop serious clinical diseases, resulting in a mortality rate of about 30%, especially in children with higher mortality rates. Even surviving infected patients mostly suffer from permanent neurological disorders, including epilepsy, paralysis, and intellectual disability. The interactions between the host and these closely related viruses further complicate the pathogenesis of this infectious disease (3, 4). JEV is a single-stranded, positive-sense RNA virus with a genome that encodes a polyprotein precursor, leading to the production of structural proteins (C, prM, and E) and nonstructural proteins (NS1, NS2A, NS2B, NS3, NS4A, NS4B, and NS5) (5). Among these proteins, NS5 plays a crucial role as the viral RNA-dependent RNA polymerase (RdRp) while also subverting STAT2-mediated antiviral interferon signaling of host innate immunity (6). In addition to its RdRp activity, the presence of methyltransferase functions confers it the status of the largest (approx. 100 kDa) and most conserved protein within the multiple JEV NS proteins (7). Furthermore, the significance of post-translational modifications (PTMs) at specific residues in *Flavivirus* NS5 protein is increasingly acknowledged as a pivotal mechanism employed by the virus to enhance viral replication (8, 9).

Several studies have implicated that the conjugation of small ubiquitin-related modifiers (SUMO) to viral NS5 protein is recognized to be evolutionarily conserved among flaviviruses, serving to enhance virus replication and suppress host antiviral response (10). SUMOylation is the process in which SUMO modification undergoes reversible covalent binding to lysine residues of the target protein (11, 12). Among the four identified SUMO isoforms in mammals, namely SUMO1 to SUMO4, SUMO2 and SUMO3 display a high sequence identity of 97% and are commonly designated as SUMO2/3 (13). In contrast, there are structural and charge distribution differences between SUMO1 and SUMO2/3, resulting in distinct functions. This process is mediated by a series of enzymes, including heterodimeric SAE1/SAE2 (SUMO-activating enzyme subunit-1 and -2) E1 complex, sole E2 conjugating enzyme UBC9, and in some cases, E3 ligases (14, 15).

The Cys2/His2 (C_2_H_2_) zinc finger proteins (ZFPs) encompass a diverse category of transcription factors that possess a diverse array of functions (16, 17). Zinc finger protein 33B (ZNF33B), a Krüppel C_2_H_2_-type zinc-finger protein, is reported to be involved in the transcriptional regulation by RNA polymerase II (18). Early investigations into Krüppel-associated box (KRAB)-ZFPs primarily focused on elucidating their molecular mechanisms in transcriptional regulation (19, 20). In recent years, extensive research has delved into the physiological and pathological roles of these family members, revealing their involvement in diverse cellular processes, such as cell differentiation, embryonic development, cell proliferation, the cell cycle, apoptosis, etc. (21–24). However, little information was reported about the roles of the KRAB-ZFPs in virus replication. Through CRISPR-based functional genomic screening, our recent study has demonstrated that zinc finger protein 33B (ZNF33B) is an essential factor for Japanese encephalitis virus infection. However, the detailed functions and underlying molecular mechanisms of ZNF33B in this process remain to be fully elucidated (3). In this study, we investigate the role of ZNF33B in facilitating JEV replication. Our data demonstrated that ZNF33B interacted with JEV NS5 and enhanced its stability by recruiting HSPB1 and HSPB8 as potential mediators of the SUMOylation. The SUMOylation of NS5 stabilized the protein by reducing its ubiquitination, particularly at lysine residues 269 and 846, thereby promoting viral replication. These findings suggest that ZNF33B plays a pivotal role in JEV replication by modulating NS5 SUMOylation through HSPB1 and HSPB8 recruitment, highlighting potential targets for therapeutic intervention against JEV infection.

## RESULTS

### Involvement of ZNF33B in JEV infection

**Fig 1:**
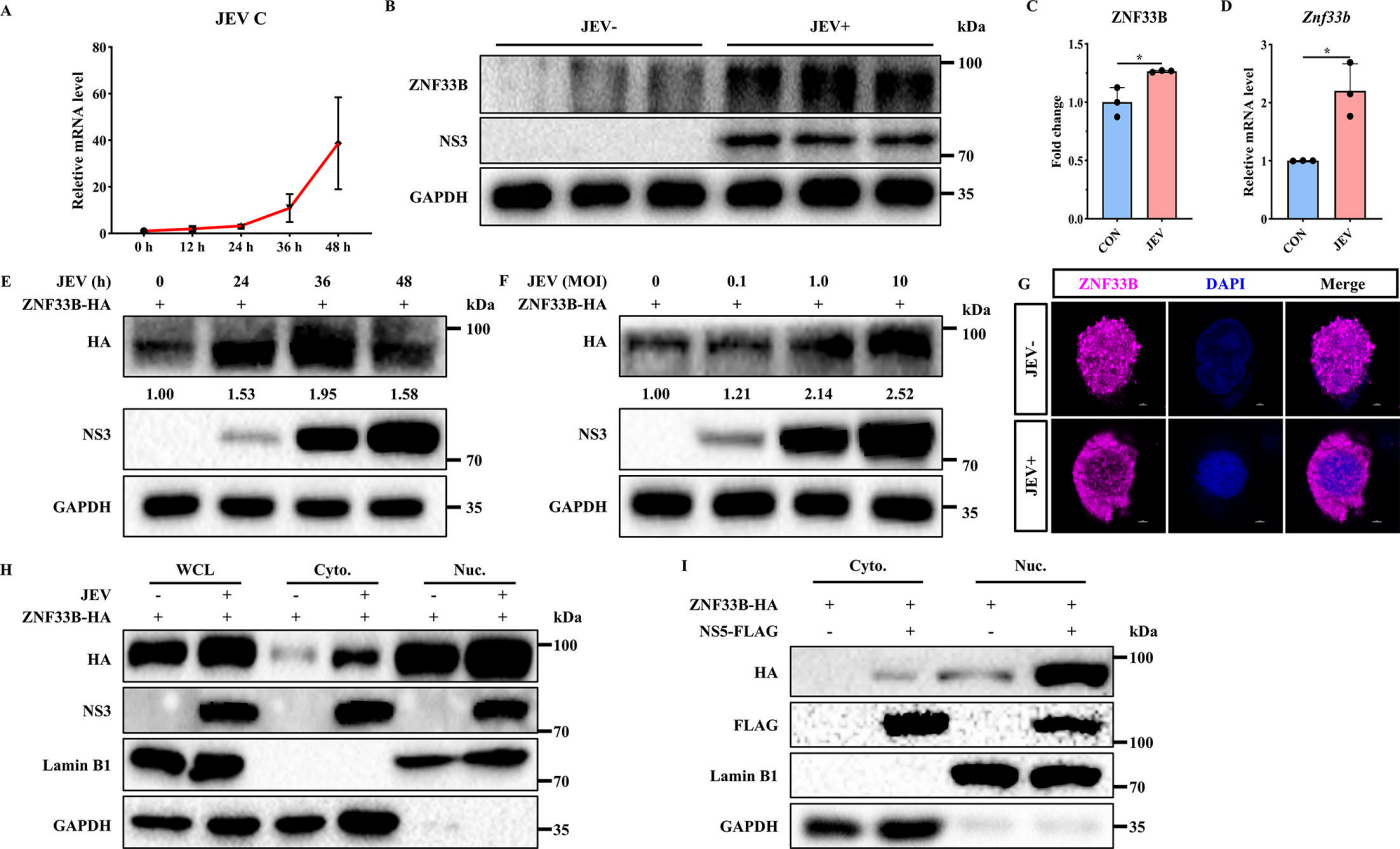
ZNF33B is implicated in JEV infection. (**A**) Quantitative PCR (qPCR) analysis of the kinetic curve of JEV replication in HEK293T cells. (**B**) Immunoblot analysis of ZNF33B expression in non-infected or JEV-infected HEK293T cells for 48 h. (**C**) The expression of ZNF33B was assessed by measuring the band grayscale with the “ImageJ” software. (**D**) The mRNA level of Znf33b was assessed by qPCR in non-infected or JEV-infected HEK293T cells for 48 h. (**E**) Immunoblot analysis of lysates from HEK293T cells transfected with ZNF33B-HA, followed by JEV infection for 24, 36, and 48 h. The expression of ZNF33B was assessed by measuring the band grayscale with the “ImageJ” software. (**F**) Immunoblot analysis of lysates from HEK293T cells transfected with ZNF33B-HA, followed by JEV infection at 0.1, 1, and 10 MOI. The expression of ZNF33B was assessed by measuring the band grayscale with the “ImageJ” software. (**G**) Confocal microscope observation of the distribution of ZNF33B in non-infected or JEV-infected cells. Scale bar, 2 µm. (**H**) Immunoblot analysis of cytoplasm and nucleus lysates from HEK293T cells transfected with ZNF33B-HA, followed by JEV infection for 48 h. (**I**) Immunoblot analysis of cytoplasm and nucleus lysates from HEK293T cells co-transfected with ZNF33B-HA and NS5-FLAG. All experiments were conducted in triplicate, and data are represented as mean ± SD. Statistical analysis was performed by a two-tailed Student’s *t*-test (*P < 0.05).

A mounting body of research has revealed the indispensable role of zinc finger proteins in regulating viral replication (25). To ascertain the role of ZNF33B in the JEV infection, we initially employed relative quantitative PCR analysis targeting the *C* gene of JEV to determine the copy number of its genome at different time points. The result demonstrated that JEV is capable of replicating in the HEK293T cells, with the highest viral multiplication rate observed between 36 and 48 h (Fig. 1A). The level of endogenous ZNF33B was subsequently assessed in JEV-infected cells, revealing a significant augmentation induced by JEV infection (Fig. 1B and C). Also, the mRNA expression of ZNF33B exhibited an increase upon JEV infection (Fig. 1D). Furthermore, we observed a significant upregulation of exogenous ZNF33B upon JEV infection at various MOIs and time points (Fig. 1E and F). Due to the nuclear localization activity of the KRAB domain (26), ZNF33B predominantly localizes within the nucleus with occasional cytoplasmic distribution (Fig. 1G). Considering that JEV replication takes place within the cytoplasmic virus replication organelles, we sought to investigate the involvement of ZNF33B in the process of JEV replication. The measurement of cytoplasm and nucleus extractions revealed that JEV infection or JEV NS5 transfection promoted the nuclear export of ZNF33B, indicating that ZNF33B might undergo translocation into the cytoplasm to participate in the JEV replication (Fig. 1H and I). Taken together, these results demonstrate that ZNF33B is involved in JEV infection.

### ZNF33B positively regulates JEV replication

**Fig 2:**
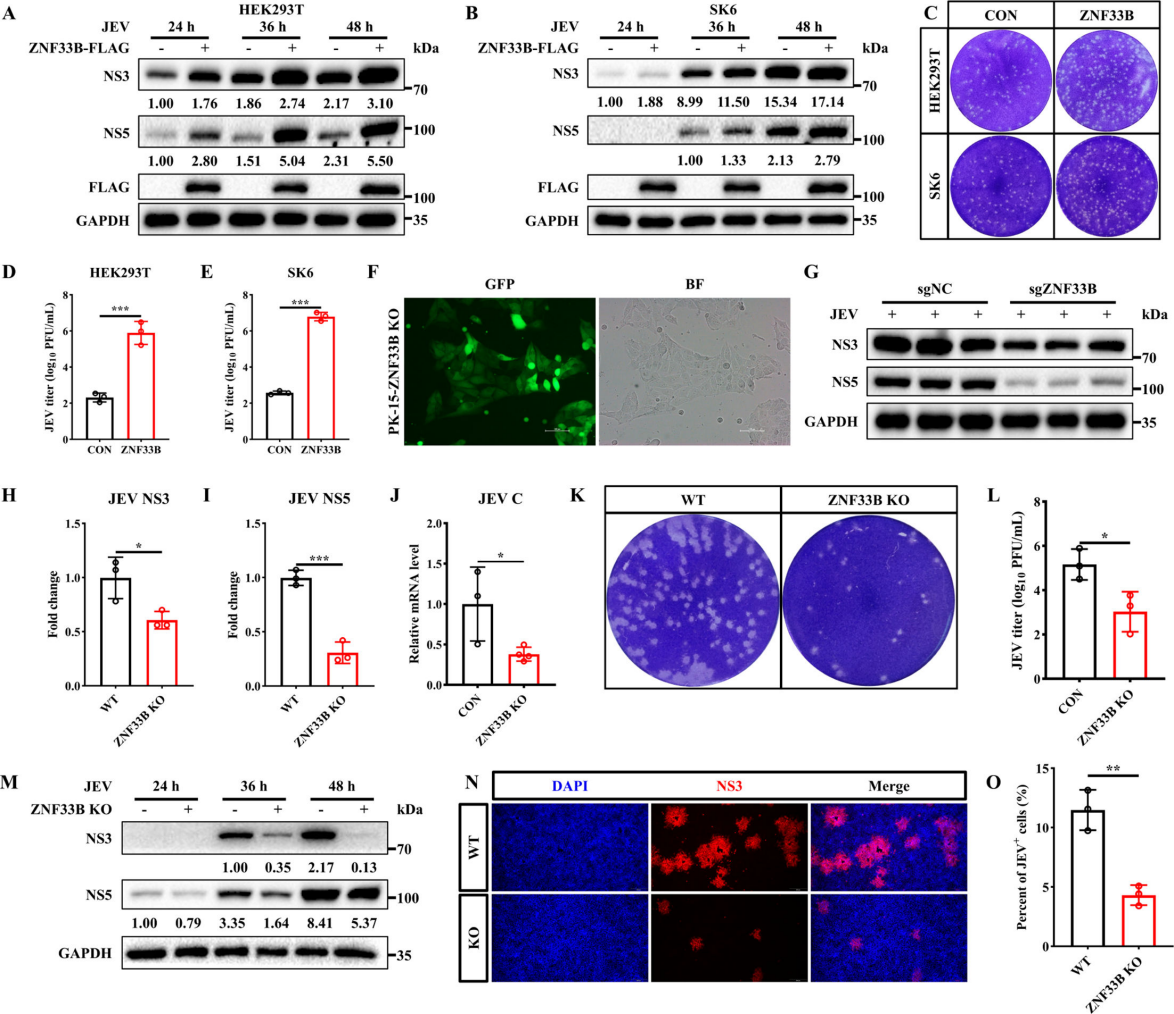
ZNF33B promotes JEV replication. (**A**) Immunoblot analysis of the expression of JEV NS3 and NS5 in HEK293T cells transfected with ZNF33B-FLAG, followed by JEV infection for 24, 36, and 48 h. The expression of JEV NS3 and NS5 was assessed by measuring the band grayscale with the “ImageJ” software. (**B**) Immunoblot analysis of the expression of JEV NS3 and NS5 in SK6 cells transfected with ZNF33B-FLAG, followed by JEV infection for 24, 36, and 48 h. The expression of JEV NS3 and NS5 was assessed by measuring the band grayscale with the “ImageJ” software. (**C–E**) The viral titration analysis of the supernatant in JEV-infected cells expressing ZNF33B was conducted by plaque assay. The statistical analysis of JEV titer in ZNF33B-overexpressed cells. (**F**) The observation on PK-15-ZNF33B KO cells by IFA. Scale bar, 100  µm. (**G–I**) Immunoblot analysis of the expression of JEV NS3 and NS5 in JEV-infected PK-15 WT and ZNF33B KO cells. The expression of JEV NS3 and NS5 was assessed by measuring the band grayscale with the “ImageJ” software. (**J**) The mRNA level of JEV C gene was assessed by qPCR in JEV-infected cells. (**K and L**) The viral titration analysis of the supernatant in JEV-infected PK-15 WT and ZNF33B KO cells was conducted by plaque assay. The statistical analysis of JEV titer in PK-15 WT and ZNF33B KO cells. (**M**) Immunoblot analysis of the expression of JEV NS3 and NS5 in JEV-infected PK-15 WT and ZNF33B KO cells for 24, 36, and 48 h. (**N and O**) The observation on JEV NS3 in JEV-infected PK-15 WT and ZNF33B KO cells by IFA. Scale bar, 100 µm. The statistical analysis of the percentage of JEV^+^ cells in PK-15 WT and ZNF33B KO cells. All experiments were conducted in triplicate, and data are represented as mean ± SD. Statistical analysis was performed by a two-tailed Student’s *t*-test (*P < 0.05, **P < 0.01, and ***P < 0.001).

Next, we investigated the potential roles of ZNF33B in JEV replication. HEK293T and SK6 cells were transfected with either an empty vector (EV) or FLAG-tagged ZNF33B plasmids, followed by JEV infection (MOI = 1) at different time points. The results demonstrated that ZNF33B significantly enhanced the levels of NS3 and NS5 proteins (Fig. 2A and B). Similarly, we also found that ZNF33B promotes the replication of JEV in human glioma U251 cells (Fig. S1A). Furthermore, supernatants from JEV-infected cells were collected at 48 h post-infection to determine viral titers using a plaque assay. Notably, we observed that viral loads increased in ZNF33B-expressing cells (Fig. 2C through E; S1B and C). Based on these findings, we then generated KO cells of ZNF33B using the CRISPR/Cas9 editing system (Fig. 2F). The PK-15 Cas9 and PK-15 ZNF33B KO cells were infected with JEV at an MOI of 1, respectively. Western blotting and quantitative PCR (qPCR) results indicated that depletion of ZNF33B induced a marked decrease in NS3 and NS5 proteins as well as JEV C mRNA level (Fig. 2G through J). Consistent with these results, the progeny virus production was also abolished in ZNF33B-depleted cells (Fig. 2K and 1L). Next, JEV replication was detected in *Znf33b*^-/-^ cells at different time points. Notably, the ZNF33B deficiency resulted in a substantial decrease in NS3 and NS5 proteins at 36 and 48 hpi (Fig. 2M). Moreover, the results of the indirect immunofluorescence assay (IFA) demonstrated a decrease in the proportion of JEV-positive cells, as indicated by reduced NS3-protein fluorescence, in *Znf33b*^-/-^ cells (Fig. 2N and O). In summary, these results suggest that ZNF33B plays a positive role in JEV replication.

### ZNF33B binds with JEV RNA to regulate its stability

Current evidence suggests that C_2_H_2_ zinc finger proteins constitute a diverse family of DNA- and RNA-binding proteins (27). Hence, we investigated whether ZNF33B plays a proviral role by associating with JEV RNA. Initially, we observed that overexpression of ZNF33B significantly augmented the JEV RNA level, while depletion of ZNF33B resulted in impaired JEV RNA level as indicated by the JEV *C* gene (Fig. S2A and B). Subsequently, the binding ability of ZNF33B with JEV RNA was assessed through RNA immunoprecipitation/qPCR assay. Notably, the JEV RNA can be immunoprecipitated by FLAG-tagged ZNF33B using anti-FLAG antibody (Fig. S2C and D). In addition, the ZNF33B-binding viral RNA pulled down by antibodies was amplified by RT-PCR with JEV 3′ UTR-specific primers. The result confirmed that ZNF33B exhibits binding affinity toward the 3′ UTR region of JEV RNA (Fig. S2E). Since JEV replication occurs in the replication complex (RC), HEK293T cells with JEV infection were processed for confocal microscopy analysis with anti-dsRNA antibody for viral RC and anti-FLAG antibody for ZNF33B protein. Co-localization of dsRNA, the intermediates of viral genome synthesis, with ZNF33B was observed at the sites of viral replication in JEV-infected cells (Fig. S2F). Given the significant impact of 3′ UTR on transcript stability, we further investigated whether the enhancement of JEV RNA level induced by ZNF33B is attributed to its regulation of viral RNA stability. The half-life of JEV RNA was assessed in JEV-infected HEK293T cells treated with actinomycin D (ActD) to suppress RNA synthesis. Our findings revealed a significantly prolonged half-life of JEV RNA in cells overexpressing ZNF33B compared to the control group (Fig. S2G). Taken together, our findings suggested that ZNF33B can bind with JEV RNA, thereby stabilizing the viral RNA to support viral replication.

### ZNF33B promotes JEV replication by interacting with NS5

**Fig 3:**
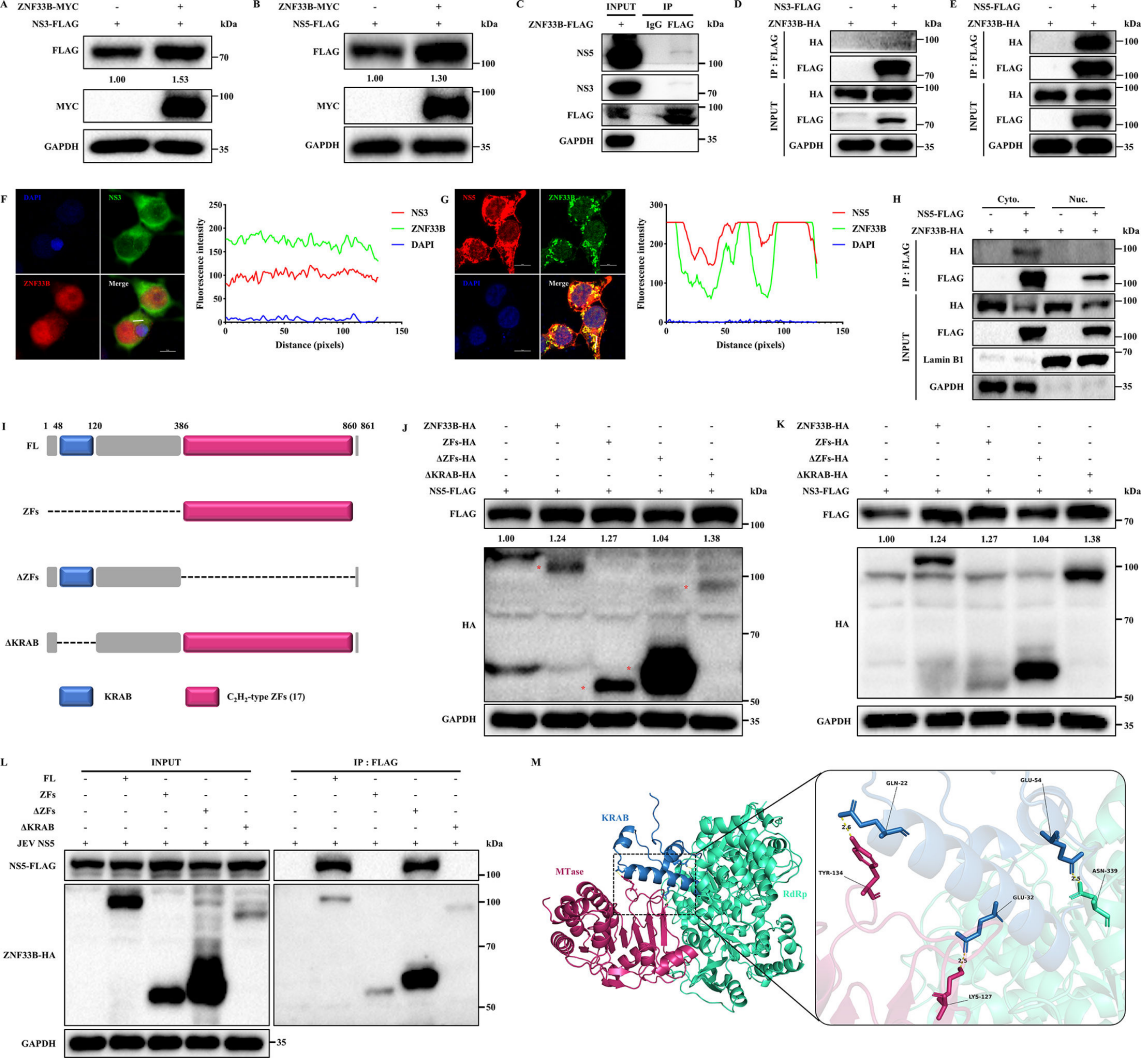
ZNF33B facilitates JEV replication by interacting with NS5. (**A and B**) Immunoblot analysis of lysates from HEK293T cells co-transfected with ZNF33B-HA and NS3-FLAG or NS5-FLAG. The expression of JEV NS3 and NS5 was assessed by measuring the band grayscale with the “ImageJ” software. (**C**) Immunoblot analysis of the association of ZNF33B with endogenous NS3 or NS5 by immunoprecipitation of lysates from HEK293T cells transfected with ZNF33B-FLAG, followed by JEV infection. The cell lysates were immunoprecipitated with anti-IgG and anti-FLAG antibodies. (**D and E**) Immunoblot analysis of the association of ZNF33B with NS3 or NS5 by immunoprecipitation of lysates from HEK293T cells co-transfected with ZNF33B-HA and NS3-FLAG or NS5-FLAG. The cell lysates were immunoprecipitated with anti-FLAG antibody. (**F and G**) Confocal microscope observation of the colocalization of ZNF33B with NS3 or NS5 in HEK293T cells co-transfected with ZNF33B-HA and NS3-FLAG or NS5-FLAG. Scale bar, 10 µm. (**H**) Immunoblot analysis of the association of ZNF33B with NS5 by immunoprecipitation of cytoplasm and nucleus lysates from HEK293T cells co-transfected with ZNF33B-HA and NS5-FLAG. The cytoplasm and nucleus lysates were immunoprecipitated with anti-FLAG antibody. (**I**) A diagram illustrating the domains of ZNF33B is presented, depicting its composition of 1 KRAB and 17 C_2_H_2_-type zinc fingers. Truncations of ZNF33B were generated, with the deletions indicated by dotted lines. (**J and K**) Immunoblot analysis of lysates from HEK293T cells co-transfected with ZNF33B-HA and its truncations, as well as NS3-FLAG or NS5-FLAG. The expression of JEV NS3 and NS5 was assessed by measuring the band grayscale with the “ImageJ” software. (**L**) Immunoblot analysis of the association of ZNF33B and its truncations with NS5 by immunoprecipitation of lysates from HEK293T cells co-transfected with ZNF33B-HA and its truncations and NS5-FLAG. The cell lysates were immunoprecipitated with anti-HA antibody. (**M**) The interaction model of the ZNF33B KRAB domain and JEV NS5 was generated by ClusPro 2.0 online software. The putative interacting amino acids were identified and annotated using PyMOL software.

Due to our findings indicating that ZNF33B can bind with JEV RNA to facilitate its replication, we focused on the interactions of NS3 and NS5, as their interaction is necessary for viral RNA synthesis. Initially, we assessed the impact of ZNF33B on the JEV NS3 and NS5 proteins by transfecting exogenous plasmids into the HEK293T cells. As anticipated, the protein levels of JEV NS3 and NS5 increased by ZNF33B overexpression (Fig. 3A and B). To elucidate how ZNF33B coordinates the expression of JEV NS3 and NS5, we tested the interaction between ZNF33B and JEV NS3 or NS5. ZNF33B exhibited a distinct interaction with endogenous JEV NS5 protein while showing a relatively minor association with JEV NS3 protein in JEV-infected cells (Fig. 3C). Reciprocal co-immunoprecipitation (Co-IP) analysis revealed a robust interaction between ZNF33B and JEV NS5 protein (Fig. 3E; Fig. S3A). However, no discernible interaction was observed between ZNF33B and JEV NS3 protein (Fig. 3D). In addition, we investigated the interactions between ZNF33B and other JEV NS proteins. The results demonstrated that ZNF33B specifically interacted with JEV NS1 and NS4B, while no interaction was detected with NS2A, NS2B, or NS4A (Fig. S3A through E). In contrast to certain *Flavivirus* NS5 proteins, JEV NS5 protein is primarily localized in the cytoplasm with a minor presence in the nucleus. Therefore, we investigated whether ZNF33B affects the subcellular distribution of JEV NS5 protein. Western blot analysis of cytoplasmic and nuclear fractions demonstrated that ZNF33B enhances both cytoplasmic and nuclear levels of NS5 protein without altering its localization (Fig. S3B). Furthermore, confocal microscopy analysis revealed the colocalization of ZNF33B with the JEV NS5 protein, rather than NS3 protein, at the perinuclear site (Fig. 3F and G). Subsequently, cytoplasmic and nuclear contents were isolated and subjected to Co-IP assay. As illustrated in Fig. 3H, ZNF33B was found to be immunoprecipitated by JEV NS5 in the cytoplasm. These findings suggest that JEV NS5 induces the nuclear export of ZNF33B to facilitate JEV NS5 expression, supporting the notion that ZNF33B may undergo translocation into the cytoplasm to participate in JEV replication (Fig. 1H and I). To further analyze the function of ZNF33B in promoting JEV NS3 and NS5, we looked at which domain is required by generating several truncations expressing the zinc finger (ZF) domain, KRAB domain deleted ZFs (ΔZFs), and ZF domain deleted KRAB (ΔKRAB) (Fig. 3I). HEK293T cells were transfected with these plasmids along with FLAG-tagged JEV NS3 and NS5, respectively, for 36 h and then harvested for western blotting analysis. Interestingly, our findings revealed that both the ZFs and ΔKRAB domains exhibited a similar promotion effect on JEV NS3 and NS5 proteins as the full-length ZNF33B. However, the ΔZF domain lost its ability to promote JEV NS3 and NS5 expression, indicating that the presence of the ZF domain is crucial for maintaining the stability of JEV NS3 and NS5 proteins (Fig. 3J and K). To determine the critical domain responsible for the interaction between ZNF33B and JEV NS5, HEK293T cells were transfected with various truncation constructs of ZNF33B along with JEV NS5-FLAG. Surprisingly, our findings indicate that it is the KRAB domain rather than the ZFs that mediates the association with JEV NS5 protein (Fig. 3L). To validate this assertion, we performed a 3D model prediction between JEV NS5 and ZNF33B KRAB. The cryoelectronic microscopy structure of JEV NS5 has been resolved previously. Unfortunately, the ZNF33B structure has not yet been resolved, but the structures of the ZNF33B KRAB domain and C_2_H_2_-type ZF domain can be predicted using AlphaFold with very high confidence according to their amino acid (aa) sequences (Fig. S3C through E). Then, the ZNF33B KRAB domain and JEV NS5 interaction model was built with ClusPro 2.0, showing that the ZNF33B KRAB domain was located in the cavity of JEV NS5 and wrapped by the MTase and RdRp domains (Fig. S3F). This model is based on computational predictions and is intended to provide a visual representation of the potential interaction interface. Next, the interaction residues between the ZNF33B KRAB domain and JEV NS5 were analyzed, and the aa residues Q22, E32, and E54 of ZNF33B KRAB could interact with the aa residues Y134, K127, and N339 of JEV NS5, respectively (Fig. 3M). Taken together, our data provide insights into the molecular mechanisms by which ZNF33B may contribute to JEV replication, particularly through its interactions with the viral NS5 protein.

### ZNF33B regulates the stability of JEV NS5 protein

**Fig 4:**
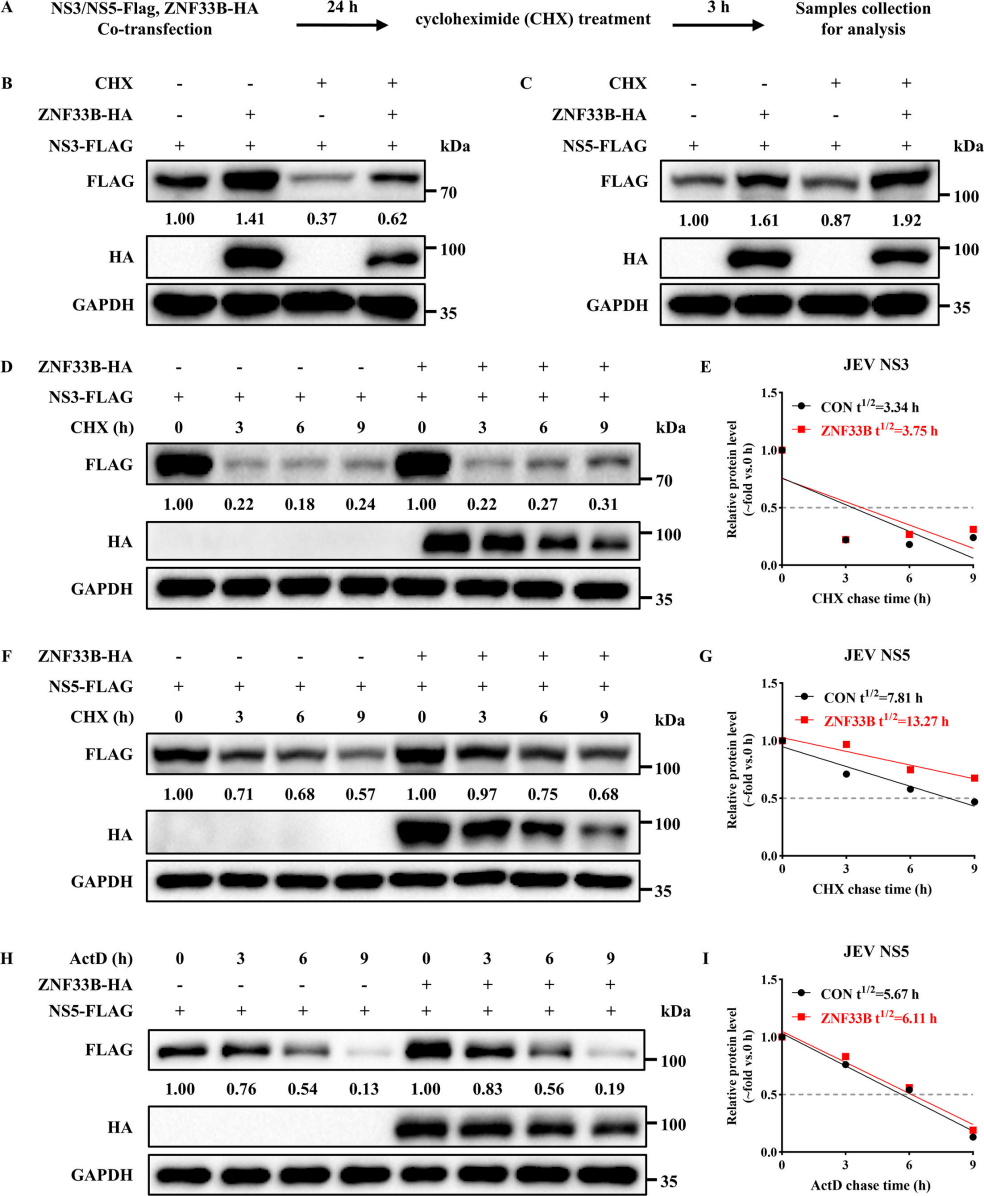
ZNF33B sustains the stability of JEV NS5 protein. (**A**) Workflow for investigating the impact of ZNF33B on the protein stability of JEV NS5. (**B and C**) Immunoblot analysis of lysates from HEK293T cells co-transfected with ZNF33B-HA and NS3-FLAG or NS5-FLAG, followed by CHX treatment for 3 h. The expression of JEV NS3 and NS5 was assessed by measuring the band grayscale with the “ImageJ” software. (**D and F**) Immunoblot analysis of lysates from HEK293T cells co-transfected with ZNF33B-HA and NS3-FLAG or NS5-FLAG, followed by CHX treatment for the indicated times. The expression of JEV NS3 and NS5 was assessed by measuring the band grayscale with the “ImageJ” software. (**H**) Immunoblot analysis of lysates from HEK293T cells co-transfected with ZNF33B-HA and NS5-FLAG, followed by ActD treatment for the indicated times. The expression of JEV NS5 was assessed by measuring the band grayscale with the “ImageJ” software. (**E, G, and I**) The protein half-life of JEV NS5 was calculated by nonlinear regression.

Although C_2_H_2_-type zinc-finger proteins are assumed to be DNA binding, some have been reported to enable the interaction with proteins to regulate proteostasis (28). Hence, we hypothesized that ZNF33B promotes the level of JEV NS5 protein by maintaining its stability. To elucidate our speculation, HEK293T cells were transfected with ZNF33B-HA and FLAG-tagged JEV NS3 or NS5 plasmids for 24 h, followed by cycloheximide (CHX) treatment to block protein translation (Fig. 4A). Consistent with our conjecture, ZNF33B rescued the decrease in JEV NS5 protein induced by CHX treatment, without affecting JEV NS3 protein (Fig. 4B and C). Subsequently, the half-life of JEV NS3 and NS5 proteins was assessed in CHX-treated cells with or without ZNF33B-HA overexpression. Our results indicated a significant extension (7.81 vs 13.27 h) in the half-life of JEV NS5 protein upon ZNF33B overexpression (Fig. 4D and E), while the half-life of JEV NS3 protein remained comparable (3.34 vs 3.75 h) to that observed in the control group (Fig. 4F and G). In addition, we investigated whether ZNF33B can regulate the expression of JEV NS5 besides protein stability. HEK293T cells were transfected with NS5-FLAG and either EV or ZNF33B-HA plasmids, followed by ActD treatment for varying durations to inhibit RNA synthesis. We observed that the degradation rate of NS5 in cells overexpressing ZNF33B was equivalent to that in the control group, with respective degradation times of 5.67 and 6.11 h, suggesting that ZNF33B may extend the half-life of JEV NS5 independently of its transcription (Fig. 4H and I). To investigate the role of the ZF domain of ZNF33B in regulating JEV NS5 stability, HEK293T cells were transfected with either ZFs or ΔZFs and JEV NS5-FLAG in the presence of CHX. Subsequently, the half-life of NS5 was assessed, revealing an extension from 8.18 to 16.22 h when the ZF domain was present (Fig. S5A and E). The ΔZF domain, however, lost the ability to attenuate the degradation of JEV NS5 protein (Fig. S5B and F). Similarly, both ZFs and ΔZFs failed to rescue the JEV NS3 protein in CHX-treated cells (Fig. S5C, D, G, and H). To rule out the impact of ZNF33B on the mRNA stability of JEV NS5, the half-life of NS5 mRNA in the presence or absence of ZNF33B was determined. The qPCR results showed that the calculated rate of mRNA decay of NS5 mRNA was comparable between the two groups, indicating that ZNF33B does not affect the stability of NS5 mRNA (Fig. S5I). These findings suggest that ZNF33B exerts a specific role in sustaining the stability of JEV NS5 protein through its ZF domain.

### ZNF33B inhibits K63-linked polyubiquitination of JEV NS5

Numerous studies have shown that the polyubiquitination of virus nonstructural proteins regulates the process of viral replication (29, 30). However, little is known about the JEV NS5 ubiquitination. Hence, we wonder whether JEV NS5 is ubiquitinated for degradation. HEK293T cells were transfected with FLAG-tagged JEV NS5, followed by CHX and MG132 treatment for 6 h. Fittingly, the level of JEV NS5 protein was reduced by treatment with CHX, while MG132 recovered its protein expression, indicating JEV NS5 undergoes ubiquitination-mediated degradation (Fig. S6A). To confirm that JEV NS5 protein can be ubiquitinated, expression plasmids for ubiquitin and its mutants retaining only a single lysine residue, including K48 and K63, were transfected into HEK293T cells together with NS5-FLAG for immunoprecipitation and immunoblot analysis. The results demonstrated that JEV NS5 protein can be ubiquitinated with a significant K48 and K63-linked polyubiquitination, suggesting that the stability of JEV NS5 protein decreased, possibly due to K48 and K63-linked polyubiquitination (Fig. S6B). Then, we explored the impact of ZNF33B on the specific ubiquitin linkage type. The results demonstrated that the WT and K63-linked polyubiquitination of JEV NS5 protein was significantly suppressed by ZNF33B (Fig. S6C through E). Taken together, ZNF33B enhanced the stability of JEV NS5 protein by inhibiting the ubiquitin-linked degradation.

### ZNF33B promotes SUMOylation of JEV NS5

**Fig 5:**
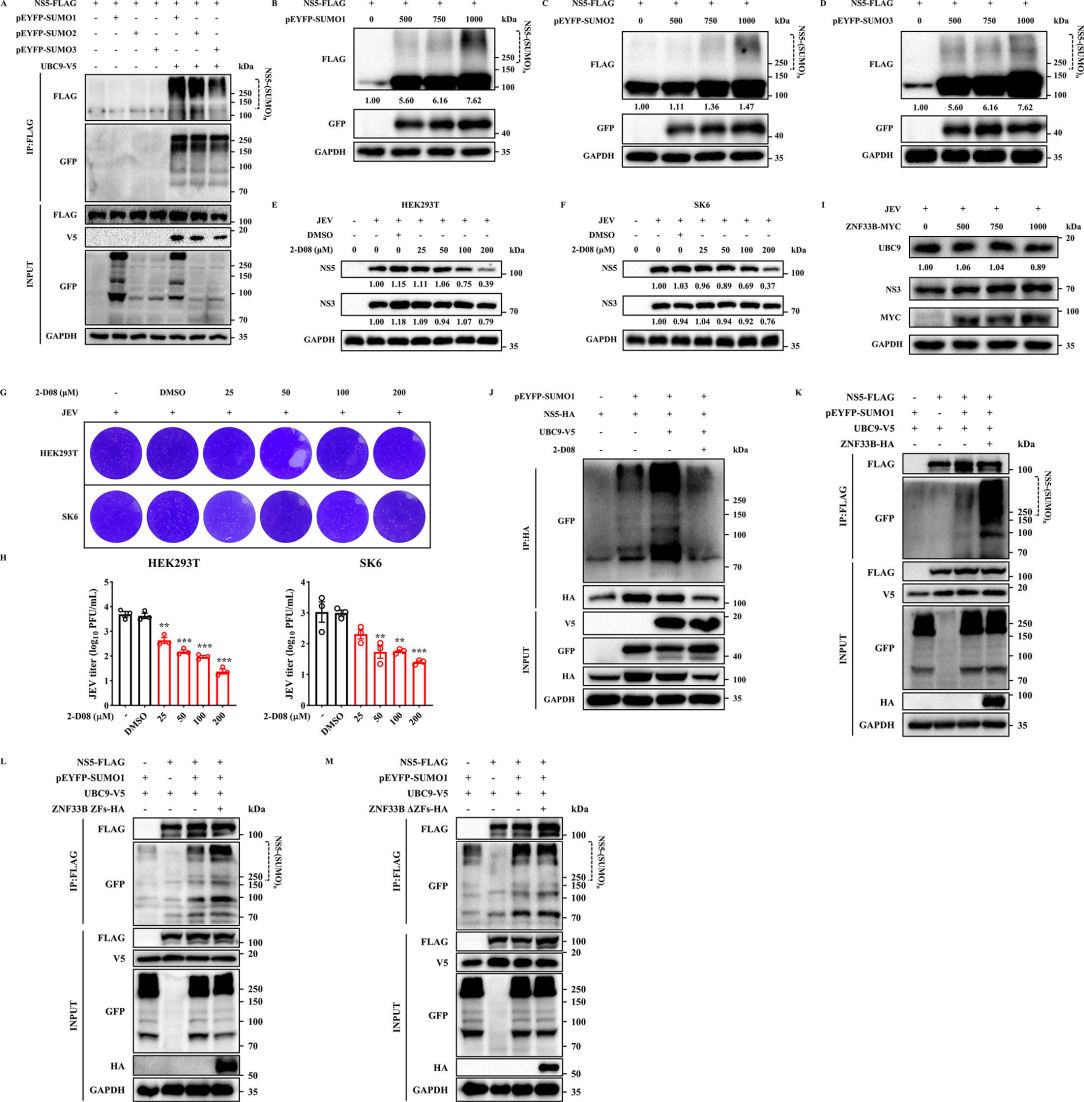
ZNF33B enhanced SUMO conjugation to JEV NS5. (**A**) Immunoblot analysis of JEV NS5 SUMOylation from HEK293T cells co-transfected with UBC9-V5, NS5-FLAG, and pEYFP-SUMO1, pEYFP-SUMO2, or pEYFP-SUMO3. The lysates were subjected to precipitation using anti-FLAG antibodies for the enrichment of SUMOylated proteins, followed by probing with the specified antibodies. (**B–D**) Immunoblot analysis of lysates from HEK293T cells co-transfected with NS5-FLAG and pEYFP-SUMO1, pEYFP-SUMO2, or pEYFP-SUMO3. The expression of NS5 was assessed by measuring the band grayscale with the “ImageJ” software. (**E and F**) Immunoblot analysis of lysates from HEK293T and SK6 cells infected with JEV, followed by DMSO or the SUMOylation inhibitor 2-D08 (0–200 μM) treatment for 6 h. (**G and H**) The viral titration analysis of the supernatant in JEV-infected cells treated with 2-D08 was conducted by plaque assay. The statistical analysis of JEV titer in 2-D08-treated cells. (**I**) Immunoblot analysis of UBC9 in HEK293T cells transfected with ZNF33B-MYC, followed by JEV infection. The expression of ZNF33B was assessed by measuring the band grayscale with the “ImageJ” software. (**J**) Immunoblot analysis of lysates from HEK293T cells co-transfected with NS5-FLAG, pEYFP-SUMO1, and UBC9-V5, followed by treatment with 2-D08 (200 µM). The lysates were subjected to precipitation using anti-HA antibodies for the enrichment of SUMOylated proteins and subsequently probed with specific antibodies to confirm the conjugation of SUMO1 to NS5. (**K–M**) Immunoblot analysis of the effect of ZNF33B and its truncations on JEV NS5 SUMOylation from HEK293T cells co-transfected with pEYFP-SUMO1, UBC9-V5, NS5-FLAG, and ZNF33B-HA, ZNF33B ZFs-HA, or ZNF33B ΔZFs-HA. The lysates were subjected to precipitation using anti-FLAG antibodies for the enrichment of SUMOylated proteins, followed by probing with the specified antibodies. All experiments were conducted in triplicate, and data are represented as mean ± SD. Statistical analysis was performed by one-way ANOVA followed by Tukey’s *post hoc* test (**P < 0.01 and ***P < 0.001).

The ubiquitination pathway, a dynamic enzymatic cascade, has been reported to be frequently modulated by various post-translational modifications, including SUMOylation and phosphorylation (31). The SUMOylation of the substrate can hinder its ubiquitination process by targeting specific lysine residues, competing for the same lysine site, and interfering with the interaction between the substrate and E3 ubiquitin ligase (32). Several studies have implicated that the conjunction of SUMO to viral NS5 protein is recognized to be evolutionarily conserved among flaviviruses and SUMOylation fine-tunes flavivirus NS5 protein stability to support its replication (10, 33); hence, we asked whether JEV NS5 protein could be modified by SUMOs. The results demonstrated that JEV NS5 can indeed undergo SUMOylation, with UBC9 being responsible for the SUMOylation of NS5 (Fig. 5A). Subsequently, we investigated the biological relevance of SUMO modification of the JEV NS5 protein by assessing the pro-effects of SUMO1/2/3 *in vitro*. Notably, SUMO1 and SUMO3 exhibited a pronounced enhancement in the protein expression levels of JEV NS5, whereas SUMO2 exerted a negligible impact (Fig. 5B through D). Moreover, we explored the anti-JEV efficacy of a recently identified SUMO inhibitor, 2′,3′,4′-Trihydroxy flavone (2-D08), *in vitro* by treating JEV-infected HEK293T and SK6 cells with different dosages, thereby investigating the biological relevance of SUMO modification of JEV NS5 protein in virus replication. As depicted in Fig. 5E and F, 2-D08 treatment violently suppresses the protein expression of JEV NS5 in a dose-dependent manner, albeit having a minimal impact on JEV NS3. Subsequently, the results of viral titers in cells infected with JEV as measured at 36 hpi through the plaque assay demonstrated that the treatment with 2-D08 significantly abated the JEV infectivity (Fig. 5G and H). Similarly, confocal microscopy analysis revealed a significant reduction in the relative fluorescence intensity of JEV NS5 following treatment with 2-D08 (200 µM), irrespective of exogenous plasmid transfection or JEV infection (Fig. S7A and B). It has been reported that SUMOylation impacts the subcellular compartmentalization and distribution of modified proteins. Therefore, we investigated the impact of SUMOylation on the cellular distribution of JEV NS5 protein. Our findings indicated that JEV NS5 protein exhibited a reduction in the cytoplasm and a slight enhancement in the nucleus by 2-D08 treatment, while both cytoplasmic and nuclear JEV NS5 protein increased by SUMO1 overexpression. Additionally, we observed a higher band corresponding to NS5 protein in the cytoplasmic fraction, suggesting potential SUMOylation events occurring in this cellular compartment (Fig. S7C and D). Consistent with the reduced level of JEV NS5 induced by 2-D08, immunofluorescence assays revealed that treatment with 2-D08 led to decreased cytoplasmic JEV NS5 protein levels and a slight translocation of the protein from the cytoplasm to the nucleus (Fig. S7E and F). Also, the application of 2-D08 resulted in a reduction of SUMOylation in the JEV NS5 protein (Fig. 5J). These findings suggest that inhibition of JEV NS5 SUMOylation promotes its translocation into the nucleus, thereby impairing its ability to participate in viral replication.

It is well established that SUMOylation is accomplished by an E1-activating enzyme, the sole E2-conjugating enzyme (UBC9), and one of several SUMO E3 ligases (34). To assess the role of ZNF33B in the SUMOylation of the JEV NS5 protein, we initially examined the impact of ZNF33B on the level of UBC9 in JEV-infected cells. As depicted in Fig. 5I, the protein level of UBC9 remains unchanged by ZNF33B. Then, we investigated the impact of ZNF33B on the SUMOylation of JEV NS5 protein. Our findings showed that ZNF33B increased the SUMO1 modification of JEV NS5 protein (Fig. 5K). In accordance with these findings, we observed that JEV NS5 was SUMOylated, and ZNF33B promoted their colocalization to a greater extent in the perinuclear compartment as determined by confocal microscopy (Fig. S7G). Next, we identified the critical domain of ZNF33B for the SUMOylation of JEV NS5 protein. Our findings unequivocally demonstrated that the ZF domain significantly enhances SUMO1 modification on JEV NS5 protein, while ΔZFs lost this ability, thereby underscoring the paramount importance of the ZF domain in promoting the stability of JEV NS5 through enhancing its SUMOylation (Fig. 5L and M).

### ZNF33B controls SUMOylation of JEV NS5 at lysine residues 269 and 846

**Fig 6:**
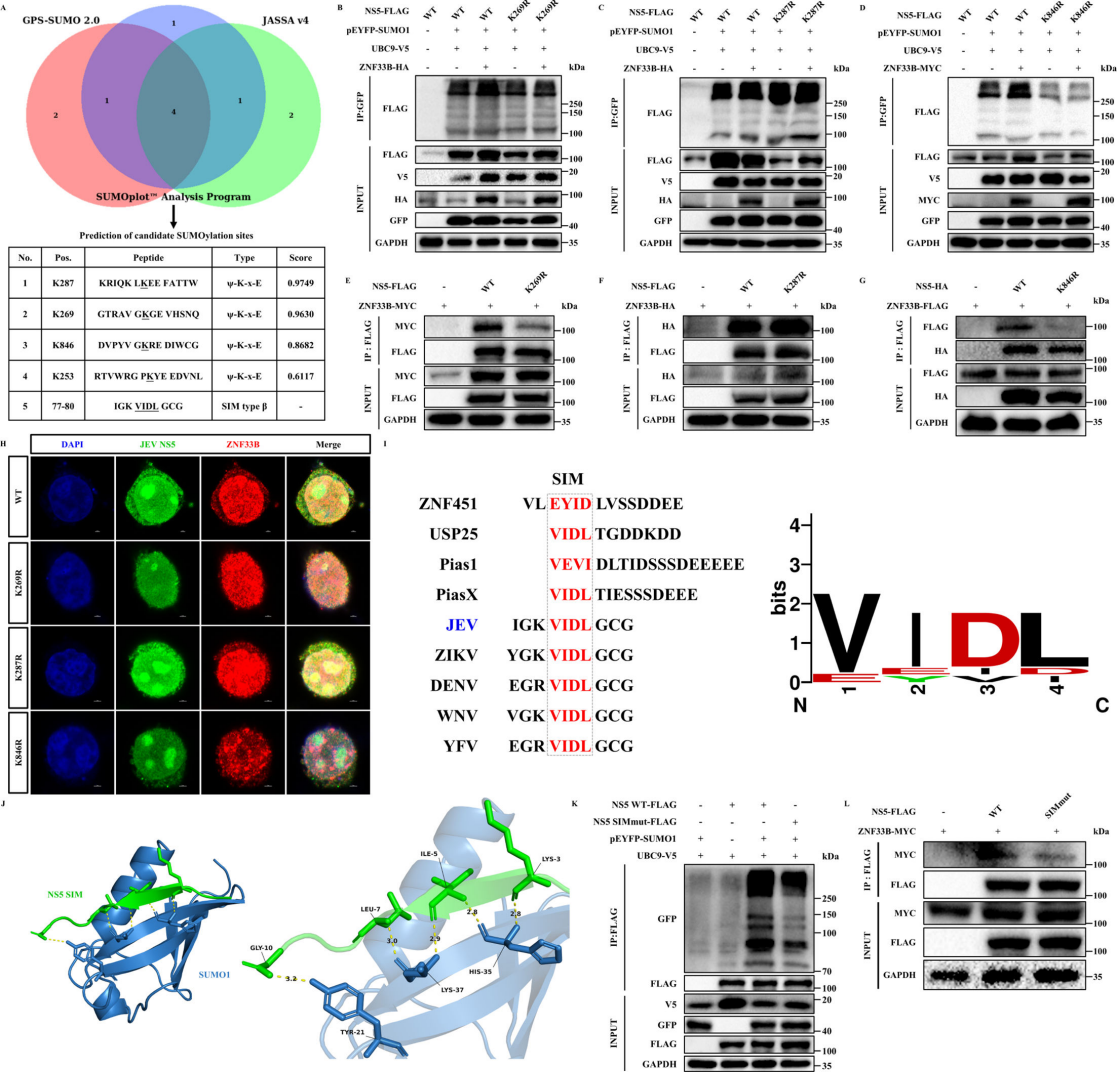
Lysine residues 269 and 846 are critical for ZNF33B-promoted NS5 SUMOylation. (**A**) The diagram illustration of SUMOylation site prediction from Advanced Services of GPS-SUMO 2.0 (https://sumo.biocuckoo.cn/online.php), JASSA version 4 (http://www.jassa.fr), and SUMOplot Analysis Program (https://www.abcepta.com/sumoplot) websites. The overlap of predicted sites is shown in the bottom table. (**B–D**) Immunoblot analysis of NS5 SUMOylation in HEK293T cells co-transfected with UBC9-V5, pEYFP-SUMO1, ZNF33B-MYC, and the WT or mutant constructs of NS5-FLAG, with arginine replacement of the lysine residues at positions 269 (**B**), 287 (**C**), and 846 (**D**). The lysates were subjected to precipitation using anti-GFP antibodies for the enrichment of SUMOylated proteins, followed by probing with the specified antibodies. (**E–G**) Immunoblot analysis of the K269R (**E**), K287R (**F**), and K846R (**G**) mutations on the association of ZNF33B with JEV NS5 by immunoprecipitation of lysates from HEK293T cells transfected with ZNF33B-MYC and NS5-FLAG. The cell lysates were immunoprecipitated with anti-FLAG antibody. (**H**) Confocal microscope observation of the colocalization of ZNF33B with the WT or mutations of NS5 in HEK293T cells transfected with ZNF33B-HA and NS5-FLAG with arginine replacement of the lysine residues at positions 269, 287, and 846. Scale bar, 2 µm. (**I**) Left, sequence alignment of SUMO interaction motifs (SIMs) derived from some E3 SUMO enzymes or flaviviruses NS5 proteins, with the dotted line indicating potential SIM. Right, a diagram depicting the SIMs generated by WebLogo (https://weblogo.berkeley.edu/logo.cgi). (**J**) The interaction model of JEV NS5 SIM and SUMO1 was generated by AlphaFold Server. The putative interacting amino acids were identified and annotated using PyMOL software. (**K**) Immunoblot analysis of JEV NS5 SUMOylation in HEK293T cells co-transfected with UBC9-V5, pEYFP-SUMO1, ZNF33B-MYC, and the WT or mutant construct of NS5-FLAG, with arginine replacement of the SIM (V-I-D-L to 4R). The lysates were subjected to precipitation using anti-FLAG antibodies for the enrichment of SUMOylated proteins, followed by probing with the specified antibodies. (**L**) Immunoblot analysis of the SIM mutations on the association of ZNF33B with JEV NS5 by immunoprecipitation of lysates from HEK293T cells transfected with ZNF33B-MYC and NS5-FLAG. The cell lysates were immunoprecipitated with anti-FLAG antibody.

To clarify the specific SUMOylation positions on the NS5 protein, the Advanced Services of GPS-SUMO 2.0 (https://sumo.biocuckoo.cn/online.php), JASSA version 4 (http://www.jassa.fr), and SUMOplot Analysis Program (https://www.abcepta.com/sumoplot) were utilized for accurate prediction of SUMOylation sites (35–37). We discovered that the lysine residues 269, 287, and 846 might be the SUMOylation sites of JEV NS5 with high reproducibility and confidence (Fig. 6A). To identify the residues modified by SUMO1, we generated single-point mutagenesis through arginine replacement of lysines on JEV NS5 and co-expressed each as a FLAG-tagged construct with SUMO1 and UBC9 in HEK293T cells. These constructs revealed that the mutation of lysine residues at 269 and 846 sites impacted NS5 SUMOylation, suggesting that these loci are crucial for the recruitment of SUMOylation machinery to JEV NS5. In keeping with this, these mutations also ablated the ZNF33B-mediated increase in NS5 SUMOylation, albeit the K287R mutation has a promoting effect (Fig. 6B through D). Subsequently, we looked into the impact of mutagenesis at the K269, K287, and K846 sites on the association between ZNF33B and NS5 *in vitro*. As expected, the interaction between ZNF33B and NS5 was abrogated by K269R and K846R, not K287R mutant (Fig. 6E through G). In line with these findings, it was observed that K269R and K846R, but not K287R mutant, displayed fewer punctate foci in association with ZNF33B (Fig. 6H).

Previous studies have demonstrated a high conservation of the putative SUMO-interaction motif (SIM) within the N-terminal domain of the NS5 protein among flaviviruses (8, 33). Subsequently, we performed sequence alignment of partial sequences encompassing the putative SIM from NS5 proteins in various flaviviruses and some E3 SUMO ligases. The amino acid sequence VIDL was identified as the putative hydrophobic core for these SIMs (Fig. 6I). To substantiate this claim, we applied the crystal structures of NS5 SIM and SUMO1 to the AlphaFold Server for molecular docking. Computational structural models were generated to predict potential interaction interfaces between NS5 SIM and SUMO1. The results confirmed that the SIM domain of NS5 interacts with SUMO1, wherein potential binding sites were identified as Lys-3, Ile-5, Leu-7, and Gly-10 of NS5 SIM corresponding to His-35, Lys-37, and Tyr-21 of SUMO1 (Fig. 6J). To ascertain the impact of NS5 SIM on its SUMOylation, a SIM mutant featuring the VIDL-to-RRRR substitution was created and subjected to an *in vivo* SUMOylation assay. The co-immunoprecipitation outcomes demonstrated that the SUMOylation was abolished in the NS5 protein harboring SIM mutation (Fig. 6K). Additionally, SIM mutation reduced the interaction between ZNF33B and JEV NS5, substantiating that ZNF33B promotes the expression level of JEV NS5 via regulating its SUMOylation (Fig. 6L).

### The SUMOylation of JEV NS5 competes with its ubiquitination

**Fig 7:**
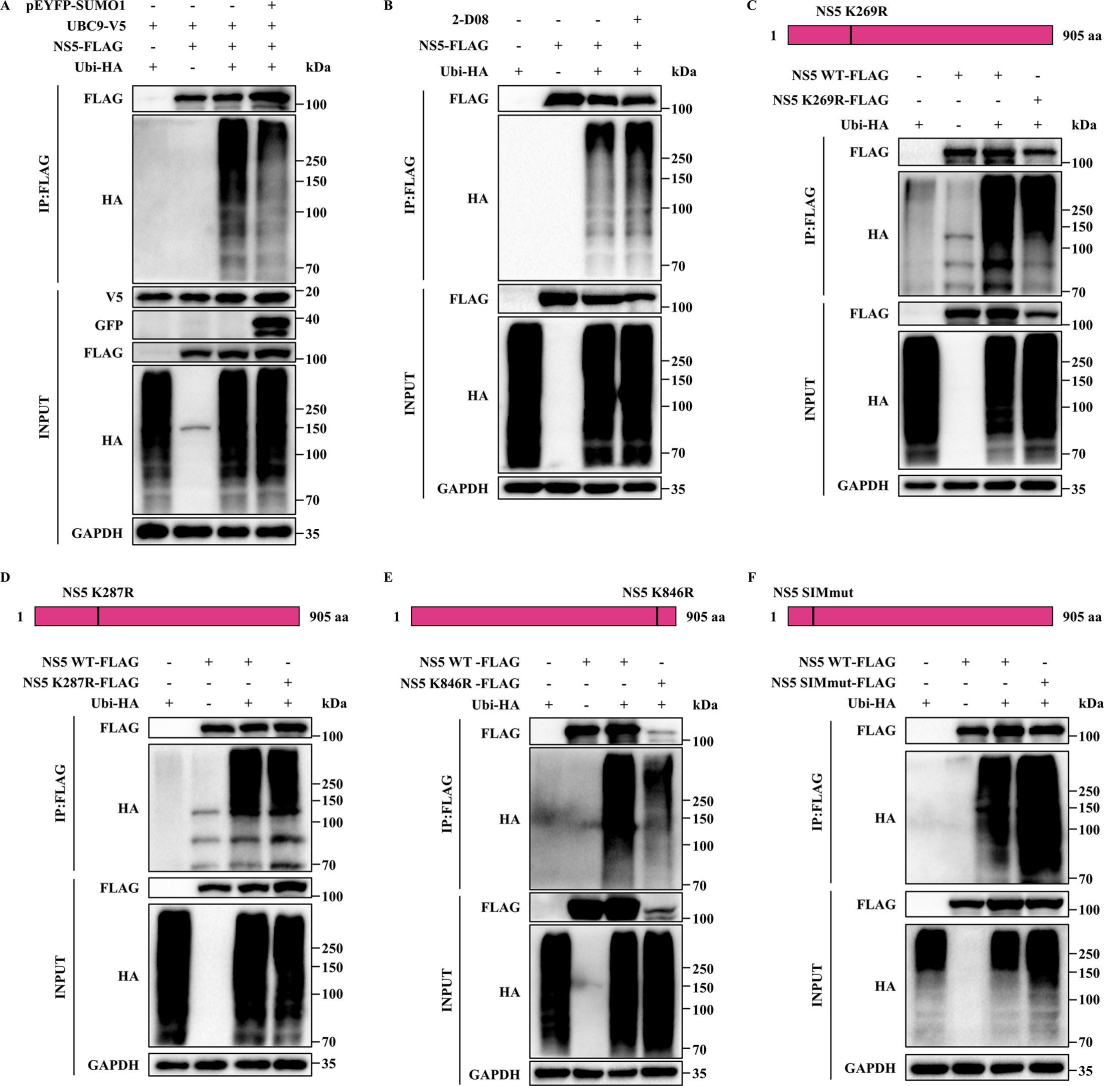
SUMOylation of JEV NS5 hinders its ubiquitination through competitive binding to lysine residues 269 and 846. (**A**) Immunoblot analysis of the effect of JEV NS5 SUMOylation on its ubiquitination in HEK293T cells co-transfected with UBC9-V5, pEYFP-SUMO1, NS5-FLAG, and Ubi-HA. The cell lysates were immunoprecipitated with anti-FLAG antibody. (**B**) Immunoblot analysis of the effect of JEV NS5 deSUMOylation on its ubiquitination in HEK293T cells co-transfected with NS5-FLAG and Ubi-HA, followed by 2-D08 (200 µM) treatment for 6 h. The cell lysates were immunoprecipitated with anti-FLAG antibody. (**C–E**) Immunoblot analysis of the K269R (**C**), K287R (**D**), and K846R (**E**) mutations of JEV NS5 on its ubiquitination by immunoprecipitation of lysates from HEK293T cells transfected with Ubi-HA and the WT or mutant constructs of NS5-FLAG, with arginine replacement of the lysine residues at positions 269 (**C**), 287 (**D**), and 846 (**E**). The cell lysates were immunoprecipitated with anti-FLAG antibody. (**F**) Immunoblot analysis of the SIM mutations of JEV NS5 on its ubiquitination by immunoprecipitation of lysates from HEK293T cells transfected with Ubi-HA and the WT or mutant construct of NS5-FLAG, with arginine replacement of the SIM (V-I-D-L to 4R). The cell lysates were immunoprecipitated with anti-FLAG antibody.

At present, the crosstalk between SUMOylation and ubiquitination has been comprehensively explored. It has been verified that SUMOylation has a substantial impact on the ubiquitination of numerous substrates (31, 38). Particularly, as SUMOylation and ubiquitination concurrently occur on the lysine residues of the substrate, it has been disclosed that SUMOylation at the same site of the substrate can, under specific circumstances, restrain its ubiquitination (32, 39). To scrutinize the crosstalk between SUMOylation and ubiquitination on JEV NS5, we analyzed the SUMOylation of JEV NS5 on its ubiquitination. The results indicated that overexpression of SUMO1 inhibited, while 2-D08 treatment promoted the ubiquitination of JEV NS5 protein, implying that the SUMOylation of JEV NS5 reduced its ubiquitination (Fig. 7A and B). In contrast to ubiquitination, which directly mediates the degradation of a protein through the proteasome pathway, SUMOylation can exert an influence on the ubiquitination of proteins by competing for the same lysine site. To gain deeper insights into the specific sites where ubiquitination and SUMOylation compete within JEV NS5, we probed into the impact of SUMOylation of NS5 on its ubiquitination at distinct lysine residues. The mutation of the primary SUMOylation sites (Lys-269 and Lys-846, not Lys-287) led to a reduction in the ubiquitination of NS5 (Fig. 7C through E). These data suggest that Lys-269 and Lys-846 may serve as competitive targets for both SUMOylation and ubiquitination. Previously, we proposed that NS5 SIM is crucial for its SUMOylation. However, it remains unclear whether NS5 SIM exerts an influence on its ubiquitination. To validate this hypothesis, we examined the impact of SIM mutation on the ubiquitination of NS5. Intriguingly, compared to the NS5 wild type, the ubiquitination level was augmented in SIM-mutated NS5 (Fig. 7F). Taken together, these results suggested that SUMOylation of JEV NS5 competitively impedes its ubiquitination at lysine residues 269 and 846.

### ZNF33B recruits HSPB1/8 to mediate JEV NS5 SUMOylation

A number of findings have suggested that the C_2_H_2_ zinc finger protein ZNF451 could be a type of SUMO E3 ligase (40). Likewise, considering ZNF33B as a C_2_H_2_ zinc finger protein that promotes the SUMOylation of JEV NS5 protein (Fig. 5K), we sought to investigate its potential role as an E3 ligase. Given that promyelocytic leukemia (PML) nuclear bodies are known hotspots by collaborating with UBC9 to enhance global cellular SUMOylation (41), our initial focus was on examining the interaction between ZNF33B and promyelocytic leukemia protein. Our findings revealed a robust interaction between ZNF33B and PML (Fig. S8A). However, contrary to our expectations, overexpression of PML inhibited JEV replication (Fig. S8B). Additionally, we noticed that ZNF33B is not associated with the sole E2 SUMO enzyme UBC9 and has no effect on UBC9 expression in JEV-infected cells (Fig. S8C and D). Therefore, we postulated that ZNF33B might recruit potential E3 ligases to regulate the SUMOylation of JEV NS5.

**Fig 8:**
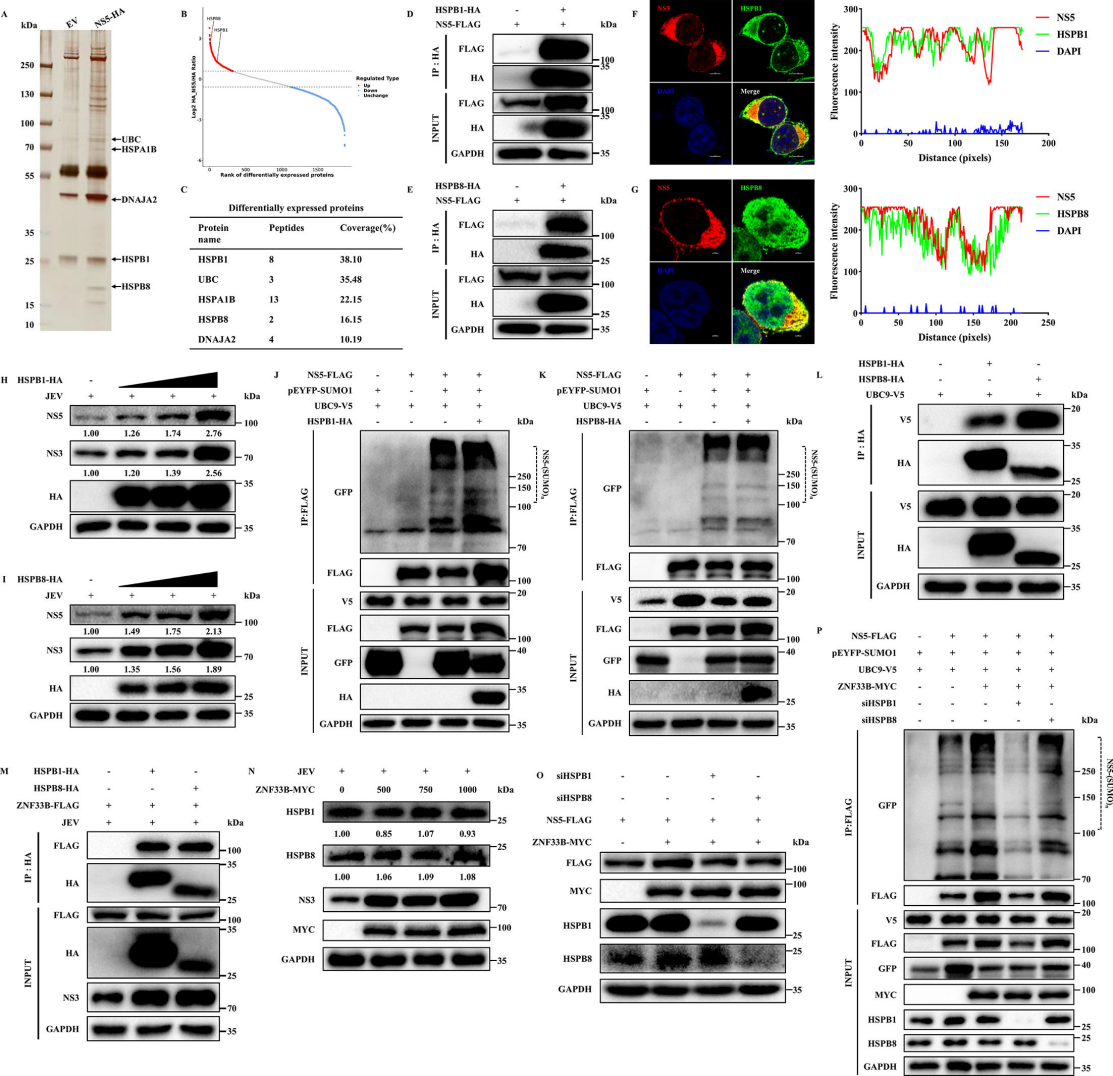
ZNF33B recruits HSPB1 to facilitate JEV NS5 SUMOylation. (**A**) Silver staining of JEV NS5-associated proteins immunoprecipitated by anti-HA antibodies probe with cell lysates from SK6 cells. The arrows indicate the additional band presented in JEV NS5-associated proteins. (**B and C**) Differentially expressed proteins analyzed by mass spectrometry. (**D and E**) Immunoblot analysis of the association of NS5 with HSPB1 or HSPB8 by immunoprecipitation of lysates from HEK293T cells transfected with NS5-FLAG and HSPB1-HA or HSPB8-HA. The cell lysates were immunoprecipitated with anti-FLAG antibody. (**F and G**) Confocal microscope observation of the colocalization of NS5 with HSPB1 and HSPB8 in HEK293T cells transfected with NS5-FLAG with HSPB1-HA or HSPB8-HA. Scale bar, 2 µm. (**H and I**) Immunoblot analysis of the expression of JEV NS3 and NS5 in HEK293T cells transfected with HSPB1-HA or HSPB8-HA, followed by JEV infection. (**J and K**) Immunoblot analysis of the effect of HSPB1 and HSPB8 on JEV NS5 SUMOylation from HEK293T cells co-transfected with pEYFP-SUMO1, UBC9-V5, NS5-FLAG, and HSPB1-HA or HSPB8-HA. The lysates were subjected to precipitation using anti-FLAG antibodies for the enrichment of SUMOylated proteins, followed by probing with the specified antibodies. (**L**) Immunoblot analysis of the association of UBC9 with HSPB1 or HSPB8 by immunoprecipitation of lysates from HEK293T cells transfected with UBC9-V5 and HSPB1-HA or HSPB8-HA. The cell lysates were immunoprecipitated with anti-HA antibody. (**M**) Immunoblot analysis of the association of ZNF33B with HSPB1 or HSPB8 by immunoprecipitation of lysates from HEK293T cells transfected with ZNF33B-FLAG and HSPB1-HA or HSPB8-HA. The cell lysates were immunoprecipitated with anti-HA antibody. (**N**) Immunoblot analysis of HSPB1 and HSPB8 in HEK293T cells transfected with ZNF33B-HA, followed by JEV infection. The expression of HSPB1 and HSPB8 was assessed by measuring the band grayscale with the “ImageJ” software. (**O**) Immunoblot analysis of the effect of HSPB1 and HSPB8 depletion on the protein level of ZNF33B-promoted NS5 in HEK293T cells transfected with ZNF33B-MYC, NS5-FLAG, and siRNAs targeting HSPB1 and HSPB8. The expression of NS5 was assessed by measuring the band grayscale with the “ImageJ” software. (**P**) Immunoblot analysis of the effect of HSPB1 and HSPB8 depletion on ZNF33B-promoted JEV NS5 SUMOylation from HEK293T cells co-transfected with ZNF33B-MYC, pEYFP-SUMO1, UBC9-V5, NS5-FLAG, and siRNAs targeting HSPB1 and HSPB8. The lysates were subjected to precipitation using anti-FLAG antibodies for the enrichment of SUMOylated proteins, followed by probing with the specified antibodies.

To investigate the E3 SUMO ligase underlying JEV NS5 SUMOylation, immunoprecipitation-mass spectrometry (IP-MS) analysis was conducted by examining proteins bound to purified EV or JEV NS5-HA in SK6 cells after sodium dodecyl sulfate-polyacrylamide gel electrophoresis (SDS-PAGE). Silver staining was employed for the detection and visualization of proteins associated with the JEV NS5 protein (Fig. 8A). After mapping with the Sus_scrofa_9823_PR_20240407 database, we obtained a total of 1,870 comparable proteins (Fig. S9A). Among these, 324 proteins showed significant upregulation, while 747 proteins exhibited significant downregulation (Fig. S9B). The mass spectrometry analysis disclosed a multitude of interacting proteins with JEV NS5, including Ubiquitin C (UBC), Heat Shock Protein (HSP) Family A Member 1B (HSPA1B), DnaJ Heat Shock Protein Family (Hsp40) Member A2 (DNAJA2), and Heat Shock Protein Beta-1 (HSPB1). Additionally, it identified a novel protein HSPB8 with a molecular weight of 27 kDa that co-purified with NS5-HA (Fig. 8B and C). To comprehend the functional targets of JEV NS5, Fisher’s exact test was employed to determine whether the differentially expressed proteins exhibit a significant tendency to enrich in the Kyoto Encyclopedia of Genes and Genomes and Gene Ontology terms. Based on this analysis, proteins bound to JEV NS5 demonstrate a prevalence in a series of biological processes and cellular signaling pathways, with a notable emphasis on heat shock protein binding and pathways related to protein processing in the endoplasmic reticulum (Fig. S9C through F). A cluster of lines suggests that HSPs are implicated in modulating the infection of numerous viruses. Additionally, it has been reported that HSPB1 functions as a SUMO-E3 ligase to regulate the SUMOylation process (42). Therefore, we selected HSPB1 and HSPB8 as candidate E3 ligases to investigate the SUMOylation of JEV NS5. We validated the interaction between JEV NS5 and HSPB1 or HSPB8 through *in vitro* Co-IP analysis. The results demonstrated a direct interaction of NS5 with both HSPB1 and HSPB8 (Fig. 8D and E). Confocal microscopy assay revealed significant colocalization of JEV NS5 with HSPB1 and HSPB8 (Fig. 8F and G). Subsequently, we explored the role of HSPB1/8 on JEV replication. HEK293T cells were transfected with HSPB1/8-HA in a dose-dependent manner, followed by JEV infection for 48 h. The results showed that HSPB1/8 enhanced the expression of JEV NS3 and NS5 proteins (Fig. 8H and I). Moreover, *in vitro* analysis revealed a significant enhancement in the abundance of JEV NS5 facilitated by HSPB1/8 (Fig. S10A and B). To further elucidate the role of HSPB1/8, we conducted small interfering RNA (siRNA)-mediated knockdown experiments. Specifically, synthesized siRNA molecules were employed to effectively suppress the endogenous expression of HSPB1/8 in HEK293T cells (Fig. S10C and D). Then, the impact of HSPB1/8 on JEV replication was ascertained by depleting HSPB1/8 in HEK293T cells, followed by JEV infection for 48 h. The results demonstrated a reduction in the protein expression of JEV NS3 and NS5 upon depletion of HSPB1/8, suggesting that HSPB1 and HSPB8 play a crucial role in facilitating JEV replication (Fig. S10E and F). Correspondingly, quantification of infectious virions through plaque assay demonstrated that the overexpression of HSPB1 and HSPB8 enhanced, while the knockdown of HSPB1 and HSPB8 suppressed JEV-induced plaque formation (Fig. S10G and H). Afterward, we investigated the impact of HSPB1 and HSPB8 on the SUMOylation level of JEV NS5 proteins by co-expressing NS5-FLAG, pEYFP-SUMO1, UBC9-V5 along with HSPB1-HA or HSPB8-HA plasmids in HEK293T cells. The results demonstrated a significant enhancement in the SUMOylation of JEV NS5 protein upon overexpression of HSPB1 and HSPB8 (Fig. 8J and K). Additionally, HSPB1 and HSPB8 exhibited a robust association and co-localization with UBC9 (Fig. 8L; Fig. S10I and J). In aggregate, our findings suggest that HSPB1 and HSPB8 may serve as crucial E3 SUMO ligases involved in mediating the SUMOylation of JEV NS5 protein. Considering that ZNF33B lacks E3 SUMO ligase activity, we aimed to investigate its potential role in recruiting HSPB1/8 to facilitate JEV NS5 SUMOylation. To this end, HSPB1/8 HA and ZNF33B-FLAG plasmids were transfected into JEV-infected cells. Our findings demonstrated that ZNF33B interacted and colocalized with both HSPB1 and HSPB8 (Fig. 8M; Fig. S10K and L), suggesting a plausible mechanism by which ZNF33B may enhance JEV NS5 SUMOylation through the recruitment of HSPB1/8. Furthermore, the effect of ZNF33B on the protein levels of HSPB1/8 was investigated. The findings revealed that ZNF33B did not induce any alterations in the protein abundances of HSPB1/8 (Fig. 8N), ruling out the possibility that ZNF33B influences the SUMOylation of JEV NS5 by modulating the expression of HSPB1/8. Therefore, we hypothesized that the HSPB1/8-mediated SUMOylation of JEV NS5 protein plays a crucial role in facilitating ZNF33B-induced stabilization of NS5. By employing two distinct siRNAs to transfect HEK293T cells overexpressing ZNF33B, we observed a significant reduction in the ZNF33B-facilitated protein level of JEV NS5 upon depletion of HSPB1/8 (Fig. 8O). In addition, the SUMOylation of JEV NS5, which is enhanced by ZNF33B, was effectively abolished upon silencing of HSPB1/8 (Fig. 8P). Taken together, our study highlights the significance of ZNF33B in facilitating the SUMOylation of JEV NS5 by the recruitment of HSPB1 and HSPB8.

## DISCUSSION

The present study unveiled a critical role for ZNF33B in facilitating JEV replication, a flavivirus with significant implications for public health. Our findings elucidated a complex interplay between host cellular factors and viral proteins, particularly focusing on the modulation of JEV NS5 protein through PTMs, specifically SUMOylation. By recruiting RNA and interacting with HSPB1/8, ZNF33B creates a favorable environment for viral replication, ensuring both the stability of viral RNA and the proper PTMs of JEV NS5.

Despite the evidence from multiple studies highlighting the involvement of KRAB-ZFPs in viral replication through targeting viral proteins, nucleic acids, and host antiviral defense mechanisms (43, 44), the report on the specific role of Krüppel C_2_H_2_-type zinc finger protein ZNF33B in viral infections remains seldom available. Our study revealed that ZNF33B undergoes translocation from the nucleus to the cytoplasm during JEV infection. Beyond its classical role in transcriptional regulation by binding to specific DNA sequences, ZNF33B could interact with JEV RNA to sustain its stability. It is widely recognized that flavivirus NS3-NS5 interaction is required for coordinated viral RNA synthesis. Intriguingly, we found that ZNF33B prolonged the half-life of JEV NS5 protein by a selective interaction with NS5, as opposed to NS3. The nuclear export of ZNF33B triggered by viral infection and subsequent interaction with viral replication complexes brought out a sophisticated strategy by the virus to hijack host factors for its replication. These findings provide new insight into a broader function of ZNF33B, not only in maintaining protein stability but also in the integrity of the viral genome during replication.

Accumulating research has highlighted the crucial roles of the host ubiquitin-proteasome system in regulating viral proteins (45). Numerous studies suggest that the ubiquitination of flavivirus viral proteins leads to various adaptations in virus replication (46). For instance, ZIKV NS1 and NS3 proteins can undergo ubiquitination for degradation by diverse functional E3 ubiquitin ligases to inhibit virus infection (47). Conversely, excessive accumulation of NS4A directly interacts with the ubiquitin E3 ligase HRD1 for ER-associated degradation to maintain optimal levels for viral replication (48). However, limited knowledge exists regarding the impact of ubiquitination on the JEV NS5 protein. Our study provides evidence that ZNF33B stabilizes JEV NS5 by inhibiting its K63-linked polyubiquitination. Through the prevention of NS5 degradation, ZNF33B ensures the sustained function of this essential viral protein, which is crucial for viral RNA synthesis and the antagonism of host antiviral responses. Protein homeostasis is meticulously maintained through a dynamic interplay of various types of PTM processes. To date, several studies have indicated that SUMOylation inhibits the ubiquitination of substrates at the same lysine residues, underscoring its significance in regulating protein stability and function (32, 49). Moreover, SUMOylation has emerged as a vital PTM in regulating viral protein stability and function (34). NS5 is one of the most conserved proteins across flaviviruses, and its SUMOylation appears to be a critical factor in JEV replication. Consequently, we deduced that the competitive modification on JEV NS5 determines the efficiency of virus replication. Our findings suggest that the competitive modification on JEV NS5 appears to govern the efficiency of virus replication, particularly through the SUMOylation-mediated hindrance of ubiquitination at lysine residues 269 and 846, thereby preventing degradation. This competitive interplay between SUMOylation and ubiquitination highlights the delicate balance involved in sustaining the stability of JEV NS5 for efficient viral RNA replication.

Interestingly, we found that ZNF33B can promote SUMOylation of JEV NS5. Although there is clear evidence indicating that several C_2_H_2_-type zinc finger proteins may be *bona fide* SUMO E3 ligases (40), we did not observe any interaction between ZNF33B and SUMO-conjugating enzymes. Through the IP-MS experiment, HSPB1 and HSPB8 were screened as candidate SUMO-E3 ligases that are recruited by ZNF33B to facilitate the SUMOylation of NS5. While the role of HSPs in viral infections is well documented as molecular chaperones aiding in protein folding and stability (50, 51), additional functions for HSPB1 and HSPB8 in the PTM of viral proteins remain poorly characterized. Our findings suggest that ZNF33B recruits HSPB1/8 to facilitate NS5 SUMOylation, which in turn reinforces the stability of NS5 and promotes viral replication, highlighting a novel mechanism by which the host cellular machinery is co-opted to benefit viral replication. Given that HSPB1 and HSPB8 are not traditionally categorized as SUMO-E3 ligases, our study also expands the functional repertoire of these proteins in viral pathogenesis.

**Fig 9:**
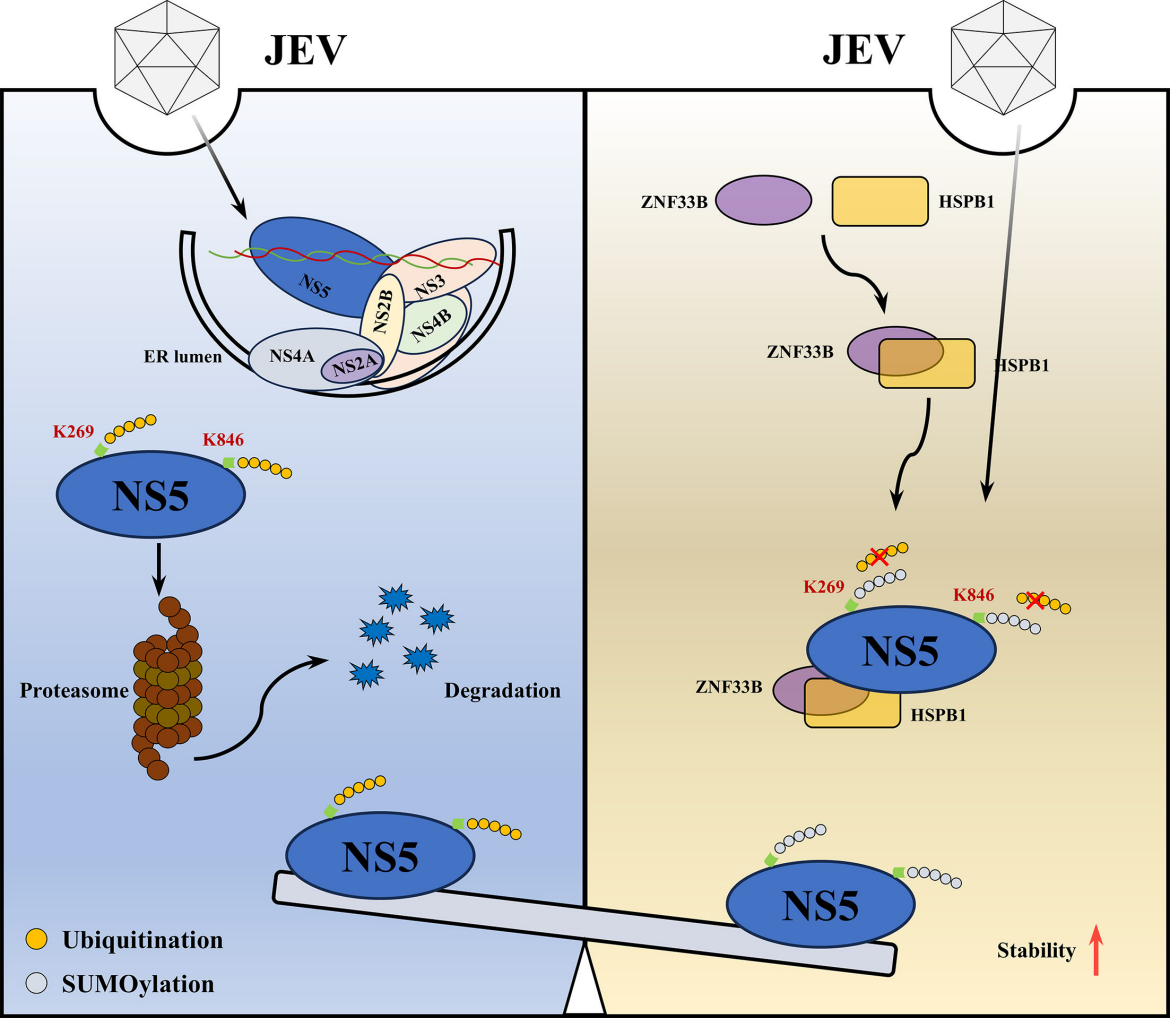
Model for the SUMOylation of JEV NS5 protein regulated by ZNF33B**.** ZNF33B facilitates Japanese encephalitis virus replication by interacting with the viral nonstructural protein 5. ZNF33B stabilizes NS5 by inhibiting its polyubiquitination and promoting its SUMOylation. The SUMOylation of NS5 competes with its ubiquitination at lysine residues 269 and 846. ZNF33B recruits HSPB1 and HSPB8 to mediate NS5 SUMOylation, which is crucial for viral replication.

In conclusion, our study reveals a novel role for ZNF33B in the replication of JEV by promoting the SUMOylation of NS5 through the recruitment of HSPB1 and HSPB8 as SUMO-E3 ligases (Fig. 9). These findings provide insights into the molecular mechanisms of JEV replication and may pave the way for the development of antiviral strategies targeting host factors and PTMs involved in viral replication.

## MATERIALS AND METHODS

### Virus and cell culture

JEV strain P3 was kindly provided by Professor Yi-ling Lin (Institute of Biomedical Sciences, Academia Sinica, Taiwan, China) and propagated in baby hamster kidney (BHK-21) cells. ZIKV was kindly provided by Wenchun Fan (Zhejiang Provincial Key Laboratory for Cancer Molecular Cell Biology, Life Sciences Institute, Zhejiang University). CSFV was stored in our laboratory. Human embryonic kidney HEK293T (HEK293T) cells and swine kidney-6 (SK6) cells were saved in our laboratory. Porcine kidney-15 (PK-15) Cas9 and ZNF33B KO cells were kindly provided by Professor Shuhong Zhao and Professor Shengsong Xie (Key Lab of Agricultural Animal Genetics, Breeding, and Reproduction of the Ministry of Education, Huazhong Agricultural University, Wuhan, China). Human glioma U251 cells were kindly provided by Professor Min Cui (State Key Laboratory of Agricultural Microbiology, College of Veterinary Medicine, Huazhong Agricultural University). All cells were incubated in Dulbecco’s modified Eagle’s minimal essential medium (DMEM; Invitrogen, USA) containing 10% fetal bovine serum (FBS; Gibco), 100 U/mL penicillin (Genview), and 10 µg/mL streptomycin sulfate (Genview) at 37°C in a humidified 5% CO_2_ incubator.

### Plasmids

cDNA encoding full-length NS1, NS2A, NS2B, NS3, NS4A, NS4B, and NS5 from JEV RP9 strain was cloned into lentiviral-based vector pTRIP-3Flag-RFP, pEGFP-C1, pCAGGS-HA, or pCR3.1-Flag as described previously. The open reading frames of specific host genes were subcloned into plasmids with epitope tags: ZNF33B-Flag, ZNF33B-MYC, ZNF33B-HA, HSPB1-HA, HSPB8-HA, and PML-HA. ZNF33B ZFs-HA, ZNF33B ΔZFs-HA, and ZNF33B ΔKRAB-HA constructs were synthesized and cloned into the pCAGGS-HA vector with N-terminal HA-tag fusion. The NS5 mutants, including K269R, K287R, K846R, and the SIM mutant, were generated from pCR3.1-NS5-FLAG plasmids through PCR-based site-directed mutagenesis. Ub-HA, K48 Ub-HA, K63 Ub-HA, pEYFP-SUMO1, pEYFP-SUMO2, pEYFP-SUMO3, and UBC9-V5 were preserved in our laboratory.

### Reagents and antibodies

Actinomycin D, cycloheximide, MG132, and DMSO were purchased from MCE (NY, USA). 2′,3′,4′-Trihydroxy flavone was purchased from Selleck (USA). The antibodies probed by ZIKV NS3 (Cat. # GTX133309), JEV NS3 (Cat. # GTX125868), and NS5 (Cat. # GTX131359) were obtained from GeneTex (USA). The antibodies probed by GAPDH (Cat. # 60004-1-Ig), Lamin B1 (Cat. # 12987-1-AP), IgG (Cat. # 30000-0-AP), FLAG (Cat. # 20543-1-AP), HA (Cat. # 51064-2-AP, 66006-2-Ig), MYC (Cat. # 16286-1-AP, 60003-2-Ig), GFP (Cat. # 50430-2-AP, 66002-1-Ig), HSPB1 (Cat. # 18284-1-AP), and HSPB8 (Cat. # 15287-1-AP) were obtained from Proteintech (USA). The antibodies probed by FLAG (Cat. # M185-6) and V5 (Cat. # M167-3) were obtained from MBL (Japan). The antibody probed by UBC9 (Cat. # HA500401) was obtained from HUABIO (China). The antibody probed by CSFV E2 was purified in our laboratory. HRP-conjugated goat anti-mouse (Cat. # 330) and HRP-conjugated goat anti-rabbit (Cat. # 458) IgG (H + L) secondary antibodies were purchased from MBL (Japan). Alexa Fluor Plus 647 Goat anti-rabbit IgG (H + L) highly cross-adsorbed secondary antibody (Cat. # A32733), Alexa Fluor 488 goat anti-mouse IgG (H + L) cross-adsorbed secondary antibody (Cat. # A32731), and Alexa Fluor 568 goat anti-rabbit IgG (H + L) cross-adsorbed secondary antibody (Cat. # A11011) were purchased from Invitrogen (USA).

### Western blotting

The cells were lysed by NP40 lysis buffer (1% NP40, 1.19% HEPES, 0.88% NaCl, and 0.04% EDTA) on ice for 2 h. After centrifugation at 12,000 rpm for 10 minutes, the protein supernatant was collected and mixed with SDS-PAGE protein loading buffer. The protein supernatant was then loaded into a 10% or 12% sodium dodecyl sulfate-polyacrylamide gel electrophoresis gel, followed by electrophoresis and wet transfer to polyvinylidene fluoride membranes (Roche, UK). Thereafter, the membranes were blocked and incubated with antibodies. The image was developed using the Bio-Rad ChemiDoc XRS + instrument and Image Lab software.

### Immunoprecipitation

The prepared protein supernatant was incubated with an appropriate amount of antibody and Protein A + G magnetic beads (Beyotime, China) at 4°C in a rolling incubator for 8–12 h. Subsequently, the mixture of protein samples and magnetic beads was subjected to magnetic separation, followed by five washes with iced NP40 lysis buffer for 10 minutes each. The elution of proteins was boiled at 95°C in 2× loading buffer for 10 minutes. The obtained proteins were utilized for western blotting assay.

### Immunofluorescence

After the implementation of appropriate treatment, cells cultured on glass coverslips were rinsed with PBS and fixed with 4% paraformaldehyde for 20 minutes, permeabilized with 0.2% Triton X-100, and blocked with 5% bovine serum albumin. Then, the cells were probed with primary antibodies, followed by exposure to fluorescent-dye-conjugated secondary antibodies. DAPI (Sigma-Aldrich, USA) was applied for nuclei staining. The images were visualized by STochastic optical reconstruction microscopy (Nikon, Japan).

### Viral titration

BHK-21 cell monolayers were incubated with serial dilutions of the cultured supernatant at 37°C for 2 h, followed by an overlay of DMEM containing 2% FBS and 3% carboxymethyl cellulose. After 4 days of incubation, viral plaques were stained with 1% crystal violet dye and counted for analysis.

### Quantitative PCR

Total RNA was extracted using the TRIzol reagent (Invitrogen, USA) and reverse transcribed into cDNA with HiScript 1st Strand cDNA Synthesis Kit (Vazyme, China) following the manufacturer’s protocol. Quantitative real-time PCR analysis was performed using the ABI StepOne Plus system (Applied Biosystems) and qPCR SYBR Green Master Mix (Yeasen, China). Specific primers are provided in Table S4. The relative mRNA expression was calculated by normalizing to GAPDH.

### RNA interference

siRNAs targeting human HSPB1 and HSPB8 were synthesized by Sangon Biotech (China) and delivered by jetPRIME transfection reagent (polyplus, USA) according to the manufacturer’s short protocol. The sequences are displayed in Table S3.

### mRNA stability

The mRNA transcription was halted by exposure to 5 µg/mL of actinomycin D (Sigma, USA). Subsequently, cells were sampled at the preconfigured time for qPCR analysis. The mRNA decay rate was measured by nonlinear regression curve fitting.

### Protein stability

HEK293T cells were transfected with the corresponding plasmids for 24 h, followed by treatment with CHX (100 µg/mL) for the indicated times. The expression of JEV NS3 and NS5 was quantified by Western blot analysis. Protein half-lives were calculated by fitting decay data to a nonlinear regression curve fitting model.

### RNA immunoprecipitation

For RNA immunoprecipitation analysis, a fraction of the cell lysate was set aside as input, while the remaining lysates were aliquoted and incubated overnight with anti-FLAG or control IgG antibodies pre-coated with Protein A + G magnetic beads (Beyotime, China) at 4°C with rotation. Subsequently, the pellets were washed using ice-cold NT2 buffer. The immunoprecipitated samples were then subjected to Proteinase K treatment (Thermo Fisher Scientific, USA) at 55°C for 20 minutes. The supernatant was collected for RNA purification and RT-PCR or qPCR analysis.

### SUMOylation assay

HEK293T cells were transiently transfected with corresponding plasmids for 24 h, followed by protein lysis using NP40 buffer. The lysates were subjected to immunoprecipitation by specific antibodies in conjunction with Protein A + G magnetic agarose (Beyotime, China). Subsequently, the beads were washed sequentially with NP40 buffer five times. Immunoprecipitates were eluted by boiling at 95°C in 2× loading buffer for 10 minutes. The target proteins and their corresponding SUMOylated species were separated by SDS-PAGE and analyzed by immunoblotting.

### Mass spectrometry

To explore the underlying proteins associated with JEV NS5, SK6 cells were transfected with JEV NS5-HA and an empty vector. Following transfection, cell lysates underwent immunoprecipitation using HA-pre-coupled magnetic beads. The immunoprecipitated proteins were then eluted, and 5 µL of suspension was used for SDS-PAGE testing. Silver staining was employed to validate the success of the immunoprecipitation process. Subsequently, the excised protein bands corresponding to NS5-HA were subjected to trypsin digestion, and the tryptic peptides were further processed for liquid chromatography-mass spectrometry analysis. The DIA data were processed using the DIA-NN search engine (version 1.8). Tandem mass spectra were searched against Sus_scrofa_9823_PR_20240407.fasta (46,176 entries), concatenated with the reverse decoy database.

### Molecular docking

The crystal structures of ZNF33B KRAB and C_2_H_2_-ZFs were generated using AlphaFold Server (52). The structures of JEV NS5 (PDB code: 4K6M) and SUMO1 (PDB code: 4JWQ) were retrieved from the Protein Data Bank (PDB). The putative NS5 SIM 10-mer peptides were separated from the crystal structures of JEV NS5 protein. The PDB files of ZNF33B KRAB and JEV NS5 were submitted to ClusPro 2.0 for binding site prediction (53). The association model of NS5 SIM and SUMO was built through AlphaFold Server. The optimal scoring model cluster was chosen for analysis and visualized using the PyMOL software.

### Statistical analysis

Statistical comparisons between two groups were conducted using the two-tailed unpaired Student’s *t*-test. For comparisons involving three or more groups, one-way ANOVA followed by Tukey’s *post hoc* test was performed to adjust for multiple comparisons. All data are presented as means ± standard deviation, and *P*-values less than 0.05 were considered statistically significant.

## Data Availability

The primary data underpinning the findings of this study are accessible within the article and its supplemental material or can be obtained from the corresponding author upon request. Source data are provided in this paper.

## SUPPLEMENTAL MATERIAL

Supplemental material jvi.00868-25-s0001.pdf Figures S1 to S10; Tables S1 to S4.

ASM does not own the copyrights to Supplemental Material that may be linked to, or accessed through, an article. The authors have granted ASM a non-exclusive, world-wide license to publish the Supplemental Material files. Please contact the corresponding author directly for reuse.
