# Supplementary material for: ZNF33B facilitates Japanese encephalitis virus replication by controlling HSPB1/8-mediated SUMOylation of nonstructural protein 5
Source: J Virol. 2025 Sep 8;99(10):e00868-25. doi: 10.1128/jvi.00868-25 (PMC12548438; doi:10.1128/jvi.00868-25)
Supplement: Supplemental material — Figures S1 to S10; Tables S1 to S4. [file jvi.00868-25-s0001.pdf]

SUPPLEMENTAL MATERIAL

ZNF33B facilitates Japanese encephalitis virus replication by controlling HSPB1/8-mediated SUMOylation of nonstructural protein 5

Jian Du[1,2,3†], Chunwei Li[1,2,3†], Jinyan Zhang[1,2,3†], Jiyuan Luo[1,2,3], Huizhi Zhang[1,2,3], Shengsong Xie[4], Huanchun Chen[1,2,3], Xiangmin Li[1,2,3,5*], Ping Qian[1,2,3,5*]

Ping Qian

Email: qianp@mail.hzau.edu.cn

This file includes:

Figures S1 to S10
Tables S1 to S4

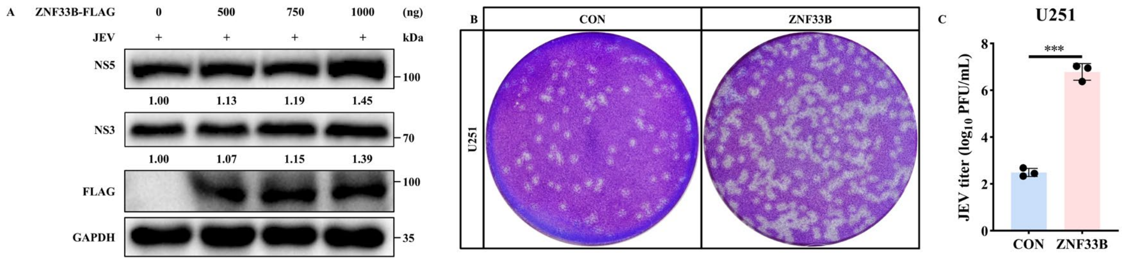

Fig S1. ZNF33B promotes JEV replication in U251 cells. (A) Immunoblot analysis of lysates from U251 cells transfected with ZNF33B-FLAG (0~1000 ng) followed by JEV infection for 48 h. The expressions of JEV NS3 and NS5 proteins were assessed by measuring the band grayscale with the "ImageJ" software. (B and C) The viral titration analysis of the supernatant in JEV-infected U251 cells expressing ZNF33B was conducted by plaque assay. The statistical analysis of JEV titer in ZNF33B-overexpressed U251 cells. All experiments were conducted in triplicate and data are represented as mean ± SD. Statistical analysis was performed by a two-tailed Student's *t*-test ($^{***}p < 0.001$).

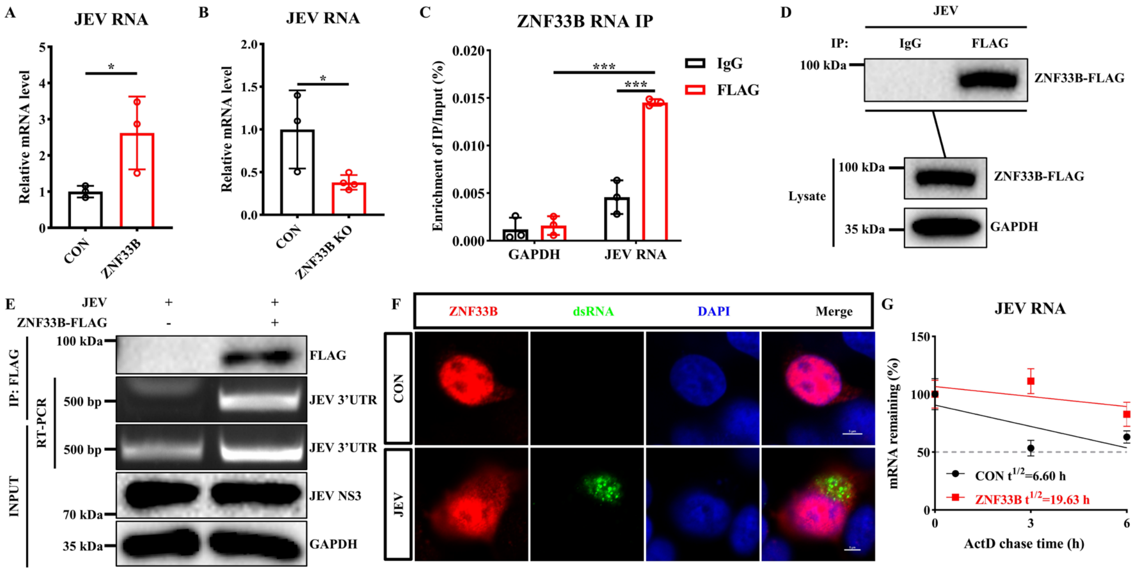

Fig S2. ZNF33B interacts with JEV RNA to prolong its half-life. (A) The mRNA level of JEV C gene was assessed by qPCR in SK6 cells transfected with ZNF33B-FLAG followed by JEV infection. (B) The mRNA level of JEV C gene was assessed in JEV-infected PK-15 WT and ZNF33B KO cells by qPCR. (C-D) The RIP-qPCR assay was performed using anti-FLAG antibody to analyze the association between ZNF33B protein and JEV RNA in HEK293T cells transfected with ZNF33B-FLAG. Immunoblot analysis of ZNF333B expression in the cell lysates and immunoprecipitated beads. (E) RIP assay using anti-FLAG antibody and RT-PCR analysis of the association between ZNF33B protein and JEV RNA with JEV 3'-UTR specific primers in HEK293T cells transfected with ZNF33B-FLAG. (F) Confocal microscope observation of the colocalization of ZNF33B and JEV dsRNA in non-infected or JEV-infected cells. Scale bar, 5 µm. (G) qPCR analysis of the level of JEV RNA in HEK293T cells transfected with ZNF33B-FLAG followed by treatment with ActD for the indicated times. The half-life of JEV RNA was calculated by nonlinear regression. All experiments were conducted in triplicate and data are represented as mean ± SD. Statistical analysis was performed by a two-tailed Student's *t*-test ($^{*}p < 0.05$ and $^{***}p < 0.001$).

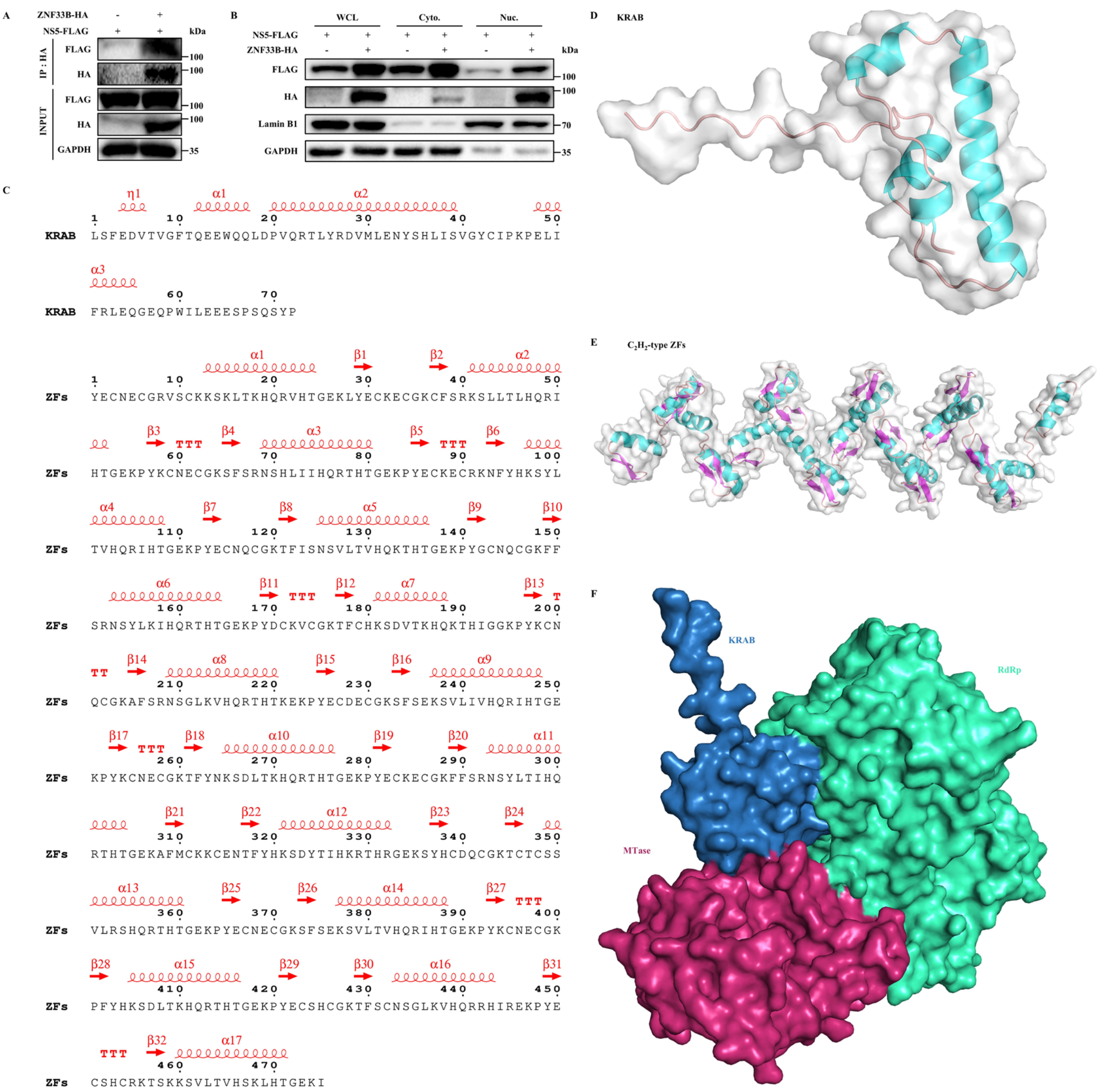

Fig S3. The interaction between ZNF33B and JEV NS5. (A) Immunoblot analysis of the association of ZNF33B with NS5 by immunoprecipitation of lysates from HEK293T cells co-transfected with ZNF33B-HA and NS5-FLAG. The cell lysates were immunoprecipitated with anti-HA antibody. (B) Immunoblot analysis of cytoplasm and nucleus lysates from HEK293T cells co-transfected with ZNF33B-HA and NS5-FLAG. (C) The secondary structures and sequences of ZNF33B KRAB and ZFs domain. The arrows indicate β-sheets and helixes indicate α-helixes. (D and E) The structural models of ZNF33B KRAB and ZFs were predicted by AlphaFold Server. (F) The interaction model of ZNF33B KRAB domain and JEV NS5 was generated by ClusPro 2.0 online software.

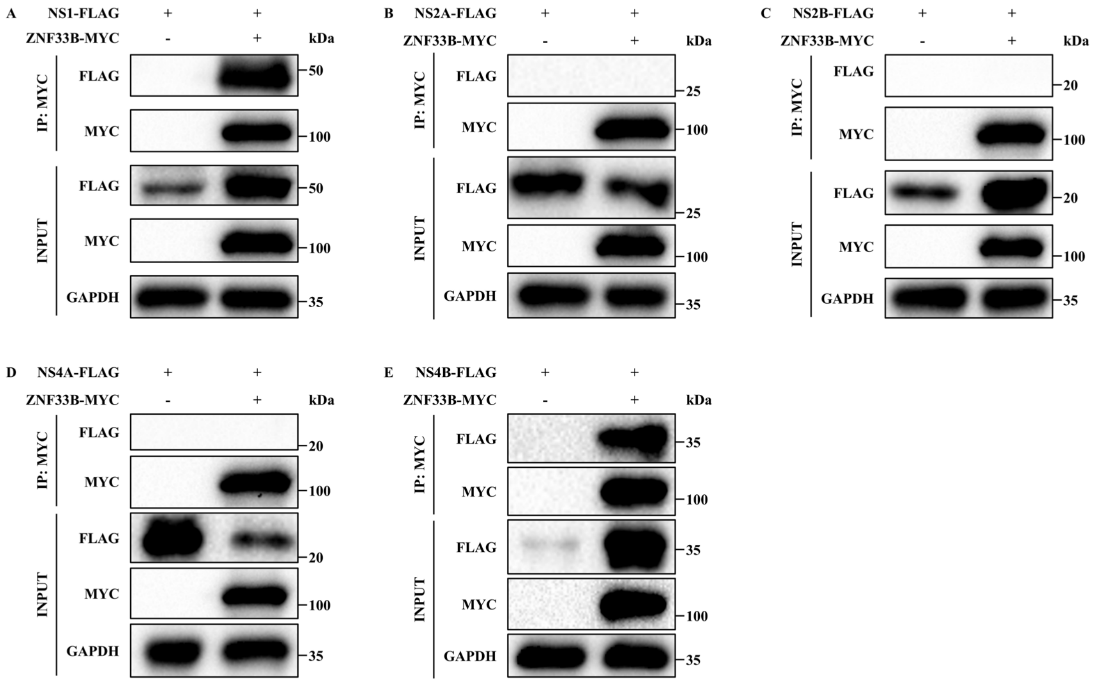

Fig S4. The interaction between ZNF33B and other JEV NS proteins. (A) Immunoblot analysis of the association of ZNF33B with NS1 by immunoprecipitation of lysates from HEK293T cells co-transfected with ZNF33B-MYC and NS1-FLAG. The cell lysates were immunoprecipitated with anti-MYC antibody. (B) Immunoblot analysis of the association of ZNF33B with NS2A by immunoprecipitation of lysates from HEK293T cells co-transfected with ZNF33B-MYC and NS2A-FLAG. The cell lysates were immunoprecipitated with anti-MYC antibody. (C) Immunoblot analysis of the association of ZNF33B with NS2B by immunoprecipitation of lysates from HEK293T cells co-transfected with ZNF33B-MYC and NS2B-FLAG. The cell lysates were immunoprecipitated with anti-MYC antibody. (D) Immunoblot analysis of the association of ZNF33B with NS4A by immunoprecipitation of lysates from HEK293T cells co-transfected with ZNF33B-MYC and NS4A-FLAG. The cell lysates were immunoprecipitated with anti-MYC antibody. (E) Immunoblot analysis of the association of ZNF33B with NS4B by immunoprecipitation of lysates from HEK293T cells co-transfected with ZNF33B-MYC and NS4B-FLAG. The cell lysates were immunoprecipitated with anti-MYC antibody.

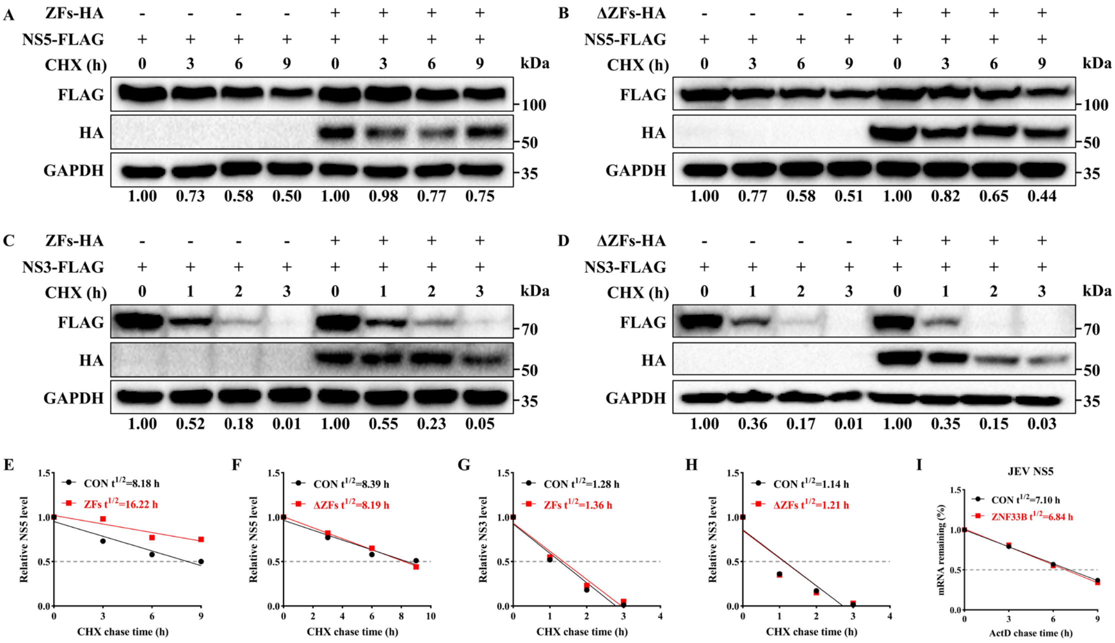

Fig S5. The ZFs domain of ZNF33B is critical for sustaining the stability of JEV NS5 protein. (A and B) Immunoblot analysis of lysates from HEK293T cells co-transfected with NS5-FLAG and ZNF33B ZFs-HA or ZNF33B ΔZFs-HA, followed by CHX treatment for indicated times. The expression of NS5 was assessed by measuring the band grayscale with the "ImageJ" software. (C and D) Immunoblot analysis of lysates from HEK293T cells co-transfected with NS3-FLAG and ZNF33B ZFs-HA or ZNF33B ΔZFs-HA, followed by CHX treatment for indicated times. The expression of NS3 was assessed by measuring the band grayscale with the "ImageJ" software. (E-H) The protein half-life of JEV NS3 and NS5 was calculated by nonlinear regression. (I) qPCR analysis of the level of JEV NS5 RNA in HEK293T cells transfected with ZNF33B-FLAG followed by treatment with ActD for the indicated times. The half-life of JEV NS5 RNA was calculated by nonlinear regression.

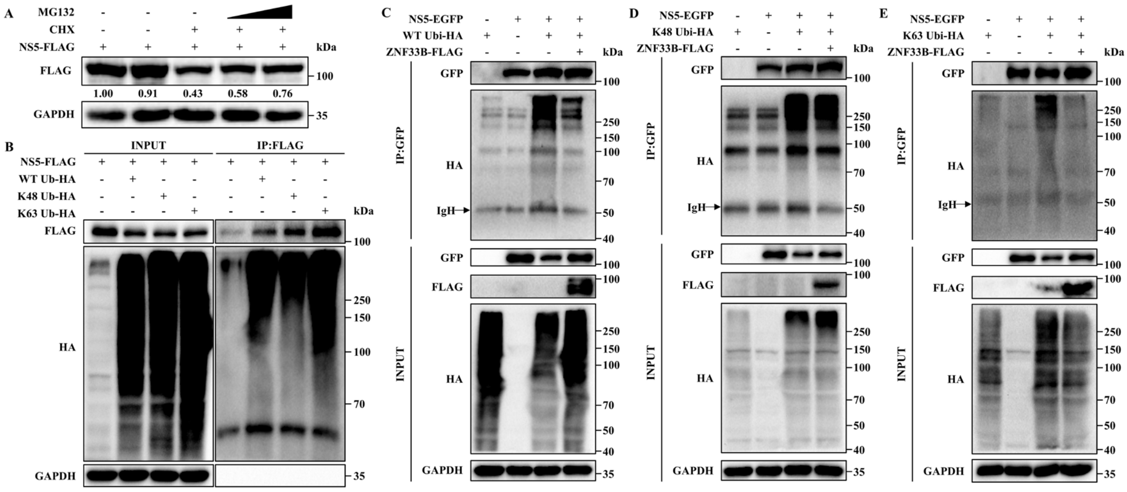

Fig S6. ZNF33B inhibits K63-linked polyubiquitination of JEV NS5. (A) Immunoblot analysis of lysates from HEK293T cells transfected with NS5-FLAG, followed by CHX and MG132 treatment for 3 h. The expression of NS5 was assessed by measuring the band grayscale with the "ImageJ" software. (B) Immunoblot analysis of JEV NS5 ubiquitination from HEK293T cells co-transfected with NS5-FLAG, and WT Ub-HA, K48 Ub-HA or K63 Ub-HA. The lysates were subjected to precipitation using anti-FLAG antibodies for the enrichment of polyubiquitinated proteins, followed by probing with the specified antibodies. (C-E) Immunoblot analysis of the effect of ZNF33B on JEV NS5 ubiquitination from HEK293T cells co-transfected with ZNF33B-FLAG, NS5-EGFP, and WT Ub-HA, K48 Ub-HA or K63 Ub-HA. The lysates were subjected to precipitation using anti-GFP antibodies for the enrichment of polyubiquitinated proteins, followed by probing with the specified antibodies.

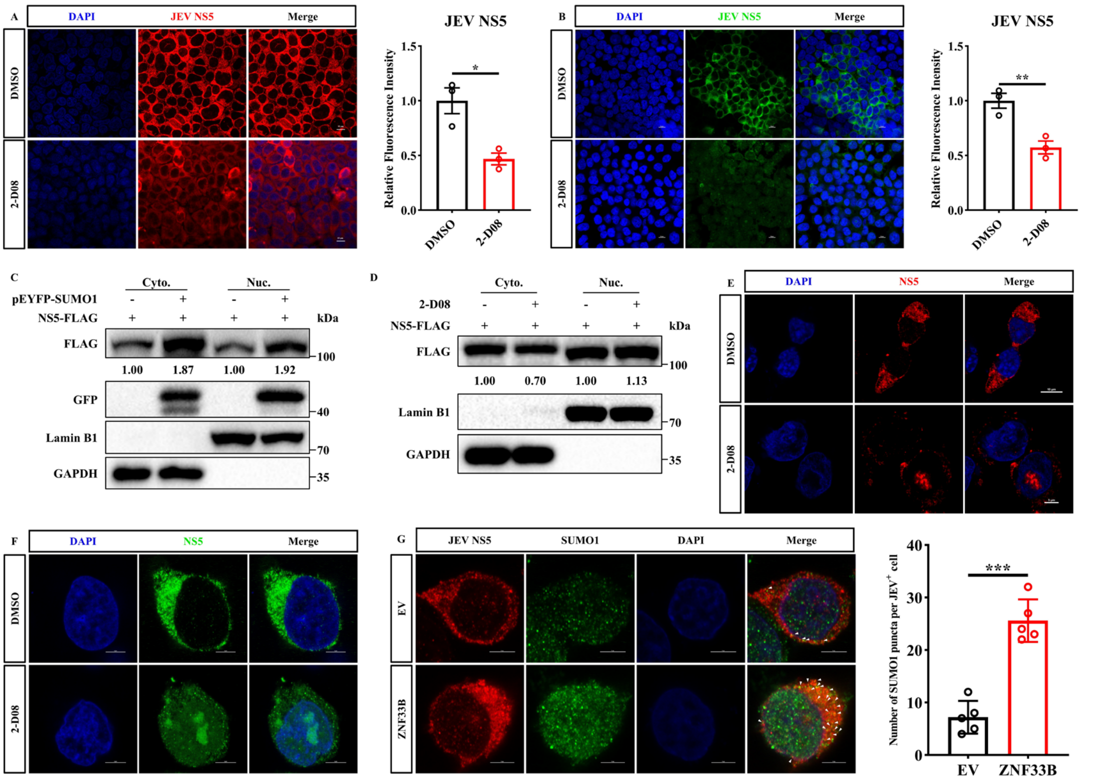

Fig S7. The SUMOylation of JEV NS5 influences its expression and subcellular distribution. (A) Left, Confocal microscope observation of the expression of JEV NS5 in HEK293T cells transfected with NS5-FLAG followed by 2-D08 (200 μM) treatment. Scale bar, 10 μm. Right, The relative fluorescence intensity of JEV NS5 was measured by the "ImageJ" software. (B) Left, Confocal microscope observation of the expression of JEV NS5 in HEK293T cells infected with JEV followed by 2-D08 (200 μM) treatment. Scale bar, 10 μm. Right, The relative fluorescence intensity of JEV NS5 was measured by the "ImageJ" software. (C) Immunoblot analysis of cytoplasm and nucleus lysates from HEK293T cells transfected with NS5-FLAG and pEYFP-SUMO1. The expression of NS5 was assessed by measuring the band grayscale with the "ImageJ" software. (D) Immunoblot analysis of cytoplasm and nucleus lysates from HEK293T cells transfected with NS5-FLAG followed by 2-D08 (200 μM) treatment. The expression of NS5 was assessed by measuring the band grayscale with the "ImageJ" software. (E) Confocal microscope observation of the subcellular distribution of JEV NS5 in HEK293T cells transfected with NS5-FLAG followed by 2-D08 (200 μM) treatment. Scale bar, 10 μm. (F) Confocal microscope observation of the expression of JEV NS5 in HEK293T cells infected with JEV followed by 2-D08 (200 μM) treatment. Scale bar, 5 μm. (G) Confocal microscope observation of the colocalization of JEV NS5 with SUMO1 in HEK293T cells transfected with ZNF33B-HA followed by JEV infection. Scale bar, 2 μm. All experiments were conducted in triplicate and data are represented as mean ± SD. Statistical analysis was performed by a two-tailed Student's *t*-test ($^{*}p < 0.05$ and $^{**}p < 0.01$).

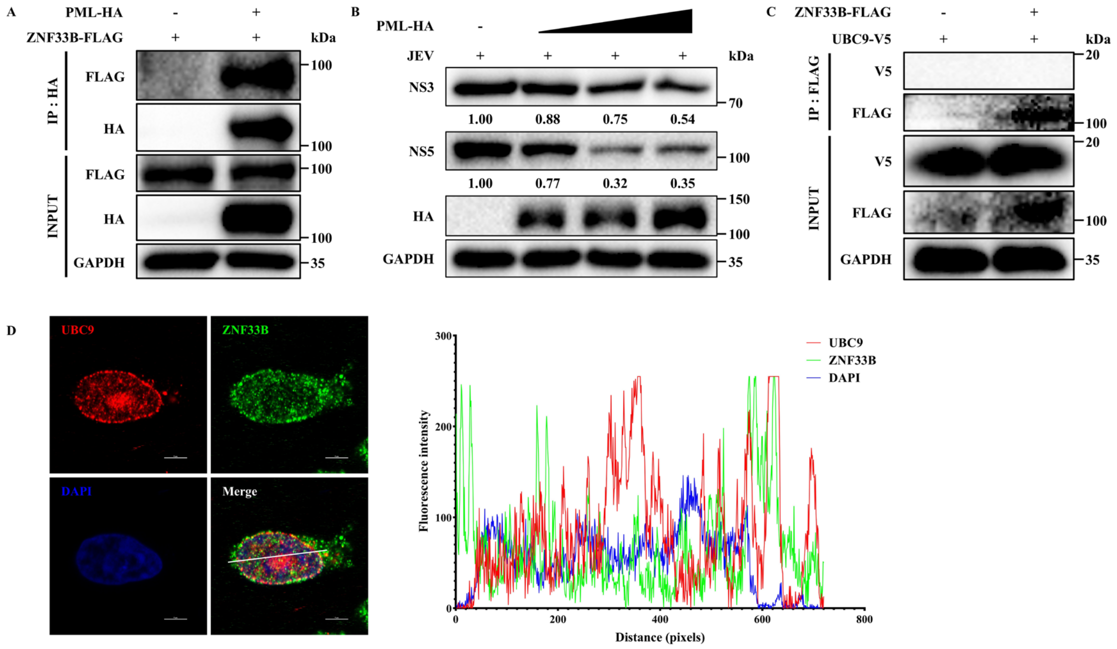

Fig S8. ZNF33B does not function as an E3 SUMO enzyme. (A) Immunoblot analysis of the association of ZNF33B with PML by immunoprecipitation of lysates from HEK293T cells transfected with ZNF33B-FLAG and PML-HA. The cell lysates were immunoprecipitated with anti-HA antibody. (B) Immunoblot analysis of the expressions of JEV NS3 and NS5 in HEK293T cells transfected with PML-HA followed by JEV infection. The expressions of NS3 and NS5 were assessed by measuring the band grayscale with the "ImageJ" software. (C) Immunoblot analysis of the association of ZNF33B with UBC9 by immunoprecipitation of lysates from HEK293T cells transfected with ZNF33B-FLAG and UBC9-V5. The cell lysates were immunoprecipitated with anti-FLAG antibody. (D) Confocal microscope observation of the colocalization of ZNF33B with UBC9 in HEK293T cells transfected with ZNF33B-HA and UBC9-V5. Scale bar, 5 μm.

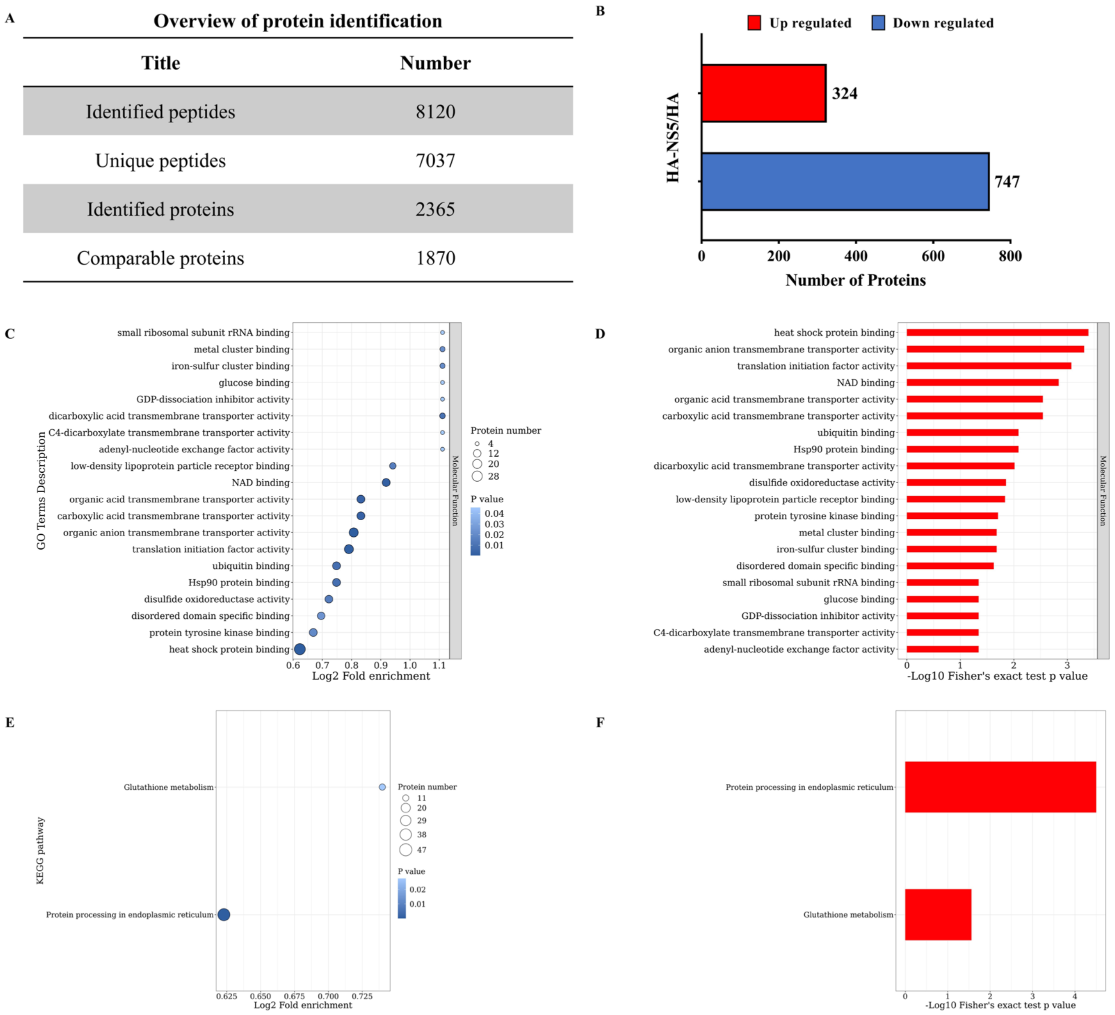

Fig S9. The functional enrichment analysis of differential protein. (A) Overview of identified proteins. (B) The number of significant differential proteins (upregulated and downregulated). (C and D) The dotplot and barplot of the molecular functions of the differential protein by Gene-ontology (GO) enrichment analysis. (E and F) The dotplot and barplot of the signaling pathways of the differential protein by KEGG enrichment analysis.

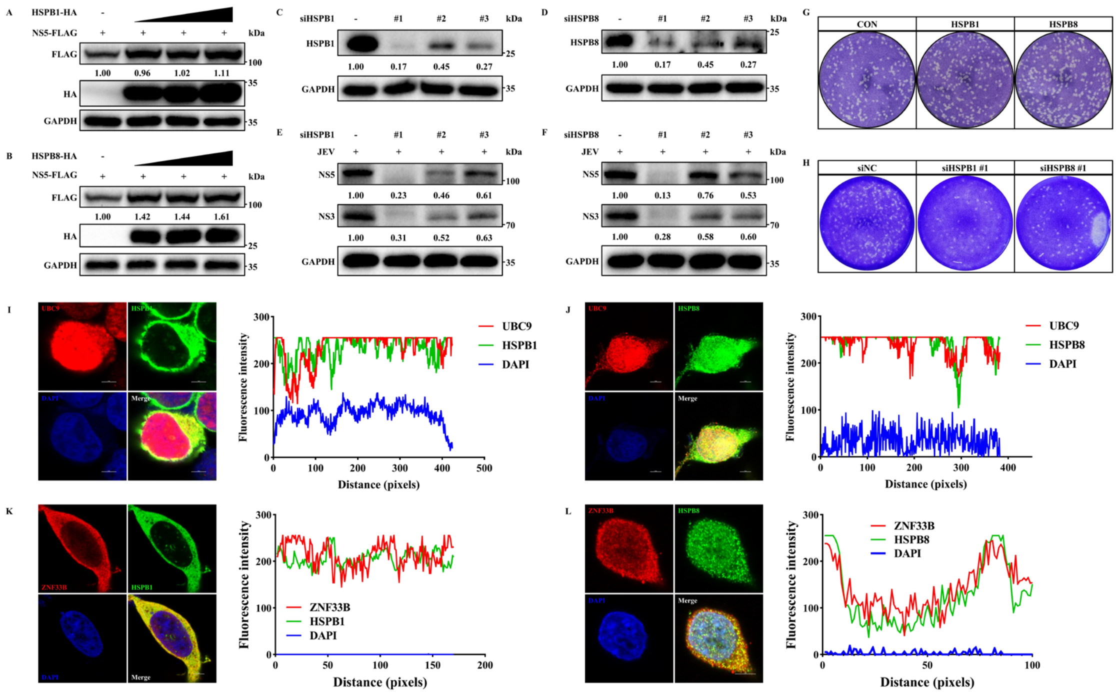

Fig S10. Identification of potential ZNF33B-associated E3 SUMO enzymes. (A and B) Immunoblot analysis of the expressions of JEV NS5 in HEK293T cells transfected with NS5-FLAG and HSPB1-HA or HSPB8-HA. The expression of NS5 was assessed by measuring the band grayscale with the "ImageJ" software. (C and D) Immunoblot analysis of the expressions of HSPB1 and HSPB8 in HEK293T cells transfected with siRNAs targeting HSPB1 or HSPB8. The expressions of HSPB1 and HSPB8 were assessed by measuring the band grayscale with the "ImageJ" software. (E and F) Immunoblot analysis of the expressions of JEV NS3 and NS5 in HEK293T cells transfected with siRNAs targeting HSPB1 or HSPB8 followed by JEV infection. The expressions of NS3 and NS5 were assessed by measuring the band grayscale with the "ImageJ" software. (G) The viral titration analysis of the supernatant in JEV-infected cells expressing HSPB1 or HSPB8 was conducted by plaque assay. (H) The viral titration analysis of the supernatant in JEV-infected cells depleted of HSPB1 or HSPB8 was conducted by plaque assay. (I and J) Confocal microscope observation of the colocalization of UBC9 with HSPB1 or HSPB8 in HEK293T cells transfected with UBC9-V5 and HSPB1-HA or HSPB8-HA. Scale bar, 5 µm. (K and L) Confocal microscope observation of the colocalization of ZNF33B with HSPB1 or HSPB8 in HEK293T cells transfected with ZNF33B-MYC and HSPB1-HA or HSPB8-HA. Scale bar, 2 µm.

Table S1 Primers employed for the gene clone

**Genes**	**Sequences (5’-3’)**
JEV NS3-F	ACTAGTATGGGTACCGCCACCATGGG
JEV NS3-R	GTCGACGAATTCTCTCTTCCCTGCTG
JEV NS5-F	GGAAGGCCTGGGGGCAGGA
JEV NS5-R	GATGACCCTGTCTTCCTGGATCAAG
JEV 3’UTR-F	AATGCATGCATATGGAGTCAG
JEV 3’UTR-R	AGATCCTGTGTTCTTCCTCAC
swine *Znf33b*-F	GAATTCATGAAGACATGTGGGTATTTT
swine *Znf33b*-R	TAAGATTTTTTCTCCTGTGTGTAATT
swine *Znf33b krab*-F	GGATCCTTATCATTTGAGGATGTGA
swine *Znf33b krab*-R	GTCGACTATCCATGGTTGCTCTCCC
swine *Znf33b zfs*-F	ATGGAAGCTTATGAATGTAATGAATG
swine *Znf33b zfs*-R	TTATAAGATTTTTTCTCCTGTGTGTAATT TTG
swine *Znf33b Δzfs*-F	ATGAAGACATGTGGGTATTTTTATCG
swine *Znf33b Δzfs*-R	TTAATCTTGTATGCATGCTCTCTTAG
swine *Hspb1*-F	ATGACTGAGCGCCGTGTGC
swine *Hspb1*-R	CTTGGTTCCGGGCTTTTCCGAC
swine *Hspb8*-F	ATGGCTGACGGTCAGATGCC
swine *Hspb8*-R	AGTACAAGTGACTTCCTGGTTGTCT
swine *Pml*-F	ATGCAGCAGGAACCGGCACCCG
swine *Pml*-R	TCAGCTCTCCTGGGAAGCCCTT

Table S2 Primers employed for the generation of JEV NS5 mutants

**Mutants**	**Sequences (5’-3’)**
K269R-F	GCCGTGGGAAGGGGAGAAGTCCATAGCAATCAGGAG
K269R-R	GACTTCTCCCCTTCCCACGGCTCTTGTTCCG
K287R-F	CAGAAGCTTAGAGAAGAATTCGCCACAACGTGG
K287R-R	GAATTCTTCTCTAAGCTTCTGGATTCTCTTCTTGATTTTCTCC
K846R-F	GTACGTGGGAAGGCGTGAGGACATCTGGTGTG
K846R-R	GTCCTCACGCCTTCCCACGTACGGAACATCTGTCCAAC
SIM (V-I-D-L to 4R)-F	ATAGGAAAACGCAGACGTCGAGGGTGTGGGCGTGGAGGATG
SIM (V-I-D-L to 4R)-R	ACGCCCACACCCTCGACGTCTGCGTTTTCCTATTGGCGAGACAAATCCTTTC

Table S3 siRNAs used for RNA interference

Genes		Sequences (5’-3’)
HSPB1	Sense #1	CGGACGAGCUGACGGUCAA/dT//dT/
	Antisense #1	UUGACCGUCAGCUCGUCCG/dT//dT/
	Sense #2	GGUGCUUCACGCGGAAAUA/dT//dT/
	Antisense #2	UAUUUCCGCGUGAAGCACC/dT//dT/
	Sense #3	GCGUGUCCCUGGAUGUCAA/dT//dT/
	Antisense #3	UUGACAUCCAGGGACACGC/dT//dT/
HSPB8	Sense #1	GAGAGAGCAGUUUCAACAA/dT//dT/
	Antisense #1	UUGUUGAAACUGCUCUCUC/dT//dT/
	Sense #2	GGUGAAGACCAAAGAUGGA/dT//dT/
	Antisense #2	UCCAUCUUUGGUCUUCACC/dT//dT/
	Sense #3	GGUGGAUCCUGUGACAGUA/dT//dT/
	Antisense #3	UACUGUCACAGGAUCCACC/dT//dT/

Table S4 primers for qPCR

Gene	Sequences (5’-3’)
JEV C-F	GAGCTTGTTGGACGGCAGAG
JEV C-R	CTCTTTTCCACTGCTTTCCATC
Znf33b-F	CGATATCGCTCAGGCTCTCC
Znf33b-R	CCATGGTTGCTCTCCCTGTT
Gapdh-F	GAGTGAACGGATTTGGCCG
Gapdh-R	CACCCCATTTGATGTTGGCG
